# Increased glycolysis in skeletal muscle coordinates with adipose tissue in systemic metabolic homeostasis

**DOI:** 10.1111/jcmm.16698

**Published:** 2021-07-06

**Authors:** Cong Xiang, Yannan Zhang, Qiaoli Chen, Aina Sun, Yamei Peng, Guoxin Zhang, Danxia Zhou, Yinyin Xie, Xiaoshuang Hou, Fangfang Zheng, Fan Wang, Zhenji Gan, Shuai Chen, Geng Liu

**Affiliations:** ^1^ State Key Laboratory of Pharmaceutical Biotechnology and MOE Key Laboratory of Model Animals for Disease Study Model Animal Research Center, School of Medicine, Nanjing University Nanjing China; ^2^ Present address: Division of Regenerative Medicine, Department of Medicine, Moores Cancer Center and Sanford Consortium for Regenerative Medicine University of California San Diego CA USA

**Keywords:** adipose tissue, FGF21, glucose sensing, glycolysis, obesity, skeletal muscle

## Abstract

Insulin‐independent glucose metabolism, including anaerobic glycolysis that is promoted in resistance training, plays critical roles in glucose disposal and systemic metabolic regulation. However, the underlying mechanisms are not completely understood. In this study, through genetically manipulating the glycolytic process by overexpressing human glucose transporter 1 (GLUT1), hexokinase 2 (HK2) and 6‐phosphofructo‐2‐kinase‐fructose‐2,6‐biphosphatase 3 (PFKFB3) in mouse skeletal muscle, we examined the impact of enhanced glycolysis in metabolic homeostasis. Enhanced glycolysis in skeletal muscle promoted accelerated glucose disposal, a lean phenotype and a high metabolic rate in mice despite attenuated lipid metabolism in muscle, even under High‐Fat diet (HFD). Further study revealed that the glucose metabolite sensor carbohydrate‐response element‐binding protein (ChREBP) was activated in the highly glycolytic muscle and stimulated the elevation of plasma fibroblast growth factor 21 (FGF21), possibly mediating enhanced lipid oxidation in adipose tissue and contributing to a systemic effect. PFKFB3 was critically involved in promoting the glucose‐sensing mechanism in myocytes. Thus, a high level of glycolysis in skeletal muscle may be intrinsically coupled to distal lipid metabolism through intracellular glucose sensing. This study provides novel insights for the benefit of resistance training and for manipulating insulin‐independent glucose metabolism.

## 1 INTRODUCTION

In the past several decades, overnutrition and physical inactivity have increased the rate of obesity worldwide, creating a global epidemic as a result of concomitant risk of diabetes, dyslipidaemia, hypertension, heart disease and certain cancers. As skeletal muscle plays critical roles in regulating whole‐body glucose homeostasis and lipid utilization, the disturbance of energy metabolism in skeletal muscle is highly relevant to the above mentioned metabolic disorders.^1^ Skeletal muscle is a major site of insulin‐mediated glucose disposal in the postprandial state. However, as the insulin signalling pathway is often disrupted in metabolic syndrome including obesity and diabetes,^2^ it is of great interest to explore insulin‐independent glucose utilizations and therapeutic options as an alternative to resolve the hyperglycaemia and to improve the systemic energy balance.

Glycolysis, although not energetically efficient, is a rapid way to consume a large amount of glucose. Aerobic exercise training has been shown to increase GLUT4 protein expression by activation of AMP‐activated protein kinase (AMPK) and Ca^2+^/Calmodulin signalling pathways while resistance training may increase GLUT1 expression, thus promoting glucose uptake in skeletal muscle irrespective of insulin.^3^ During prolonged sub‐maximal exercise, the oxidative metabolisms of lipids and carbohydrates supply most of the energy in skeletal muscle, whereas the anaerobic metabolisms of glycogen and glucose are the dominant energy‐producing pathway during short‐term high‐intensity exercise.^4^ Although aerobic and resistance training are both effective in reducing abdominal adipose mass and plasma glucose level in adults with prediabetes, resistance training, which increases the abundance and capacity of glycolytic muscles,^5^ is effective in augmenting muscle mass and increasing the basal metabolic rate.^6^, ^7^ In addition, exercise‐induced muscle adaption also contributes to metabolic regulation and health through the release of myokines in cross‐talk with the remote metabolic organs.^8^

When activated by resistance training, mechanistic target of rapamycin complex 1 (mTORC1) increases the translation of hypoxia‐inducible factor 1α (Hif1α), which drives glycolysis and pentose phosphate pathway (PPP) through elevating the expression of specific metabolic genes including *Glut, Hk, Pfk* and *G6pd*.^9^, ^10^ However, overexpression of GLUT1 alone did not alleviate diet‐induced insulin resistance and obesity in mice although prevented the development of glucose intolerance.^11^, ^12^ Moreover, although co‐expression of HK2 with GLUT1 increased the steady‐state level of glucose metabolites in muscles, this strategy did not further improve whole‐body metabolic regulation beyond that of GLUT1 overexpression in mouse muscles.^13^ Thus, the control of enhanced glycolysis in muscles as well as its link to systemic metabolic regulation is not well defined.

Organisms regulate their physiology by continuously perceiving and transmitting signals within the cell and the environment. It is well known that liver possesses multiple glucose‐sensing systems, in which the glucokinase‐ChREBP axis functions as a central node, coordinating glucose metabolism with cell signalling and gene expression to accommodate systemic fuel availability.^14^ Not only hepatocytes, recent studies indicated that skeletal myocytes could directly sense extracellular glucose via the K_ATP_ channel to activate Baf60c, which promoted the glycolytic metabolism in muscle, contributing to the metabolic improvement of diet‐induced obese mice through Deptor‐mediated AKT activation.^15^, ^16^ In a skeletal muscle‐specific Akt1 transgenic mouse, Akt1‐mediated growth of glycolytic muscle led to ameliorated obesity with improvement in insulin sensitivity and reductions in serum insulin and glucose.^17^, ^18^ It has been suggested that skeletal muscle‐secreted FGF21, regulated by an Akt1/activating transcription factor 4 (ATF4)‐dependent mechanism, coordinates these metabolic activities.^19^, ^20^ With other pathways also known to regulate glycolysis and the existence of diverse glucose‐sensing mechanisms,^21^ it will be important to determine whether enhancing glycolysis in skeletal muscle possesses an intrinsic and casual link with systemic metabolic benefit and the molecular and cellular pathways underlying such a link.

Glycolytic metabolism requires synergistic expression and activity of multiple glycolytic enzymes. GLUT1 is responsible for insulin‐independent glucose uptake, the first step of glycolysis. HK2 catalyses the first rate‐limiting step of the glycolytic pathway, where glucose is phosphorylated to G‐6‐P. Increasing PFK2 activity would lead to an increase in F‐2,6‐BP levels, thereby promoting glycolysis by activation of PFK1, the rate‐limiting enzyme in the process.^22^ As PFKFB3 has the highest kinase/phosphatase activity ratio resulting in high concentration of F‐2,6‐BP,^23^ we established a BAC transgenic mice with Muscle‐specific overexpression of three Glycolytic genes *PFKFB3‐HK2‐GLUT1* in a polycistronic manner (referred to as M;G mice) to mimic the enhanced glycolysis stimulated by resistance training and to directly interrogate the role of glycolysis of skeletal muscle in control of systemic homeostasis. We found that the enhanced anaerobic glycolytic metabolism in skeletal muscle of M;G mice could directly improve systemic metabolic profiles through metabolic coordination with adipose tissue. The cross‐organ balances between anaerobic and oxidative metabolism as well as carbohydrate and lipid metabolism found in this study provide novel insights for the benefit of anaerobic exercise and offer new ways to manipulate glycolysis for the benefit of metabolic health.

## 2 MATERIALS AND METHODS

Detailed description of methods is provided in the Supporting information.

### 2.1 Experimental animals

Mice were bred and maintained under specific pathogen‐free conditions in a controlled environment of 20‐22 ℃, with a 12/12‐h light/dark cycle and free access to food and water unless stated. Experiments involving animals were approved by the Institutional Animal Care and Use Committee (IACUC) of Model Animal Research Center, Nanjing University, China, and conducted in accordance with the guidelines of IACUC and the approved Animal protocol #LG19. Genotyping was carried out using primers listed in Table S1. All the experiments were performed in male mice, except that skeletal muscles from female mice were used for muscle glucose uptake.

### 2.2 Gene expression and protein analysis

The sequences of primers for Q‐PCR were listed in Table S2, and the antibody information was provided in Table S3.

### 2.3 Measurement of oxygen consumption in cells and tissues

Both the extracellular acidification rate (ECAR) and oxygen consumption rate (OCR) of mouse embryonic fibroblast cells (MEFs) were measured using the XF^e^ extracellular flux analyzer (Seahorse Bioscience). Mitochondrial respiration rates were measured in saponin‐permeabilized extensor digitorum longus (EDL) muscle and white fat tissue with indicated substrates.

### 2.4 Muscle glucose uptake and lipid uptake and oxidation ex vivo

Intact soleus and EDL muscles were isolated from female mice for glucose uptake, and soleus isolated from male mice were applied for lipid uptake and oxidation.

### 2.5 Glucose, insulin and oral lipid tolerance tests (GTT, ITT and OLTT)

After withdrawal of food for 16 h (for GTT) or 4 h (for ITT and OLTT), mice were intraperitoneally (i.p.) injected with a bolus of glucose (2 mg glucose per g of bodyweight) for IPGTT, insulin (0.75 mU insulin per g of bodyweight) for ITT or orally administered via gavage with a bolus of olive oil (6 ul olive oil per g of bodyweight) for OLTT. Blood was collected and measured from tail veins at the indicated times.

### 2.6 Assessment of body composition and treadmill endurance

Body composition was determined via dual‐energy X‐ray absorptiometry using a Lunar PIXImus II densitometer (GE Healthcare) following the manufacturer's instructions. For exercise studies, fed mice were run for 10 minutes at 10 m/minute followed by a constant speed of 20 m/minute until exhaustion.

### 2.7 Indirect calorimetry

Mice were housed individually in metabolic cages at a 12‐h light and dark cycle with free access to food and water using the Comprehensive Lab Animal Monitoring System (CLAMS, Columbus Instruments).

### 2.8 Metabolomics analysis

LC‐HRMS was performed on a Waters UPLC I‐class system equipped with a binary solvent delivery manager and a sample manager, coupled with a Waters VION IMS Q‐TOF Mass Spectrometer equipped with an electrospray interface (Waters Corporation).

### 2.9 Cell culture, cell line generation and RNAi experiments

C2C12 cells (ATCC^®^ CRL‐1772^™^) were cultured and differentiated according to ATCC directions. Cells were transfected with pRosa‐CAG‐rtTA‐TRE‐Transgene‐Neo plasmids using Lipofectamine 3000 transfection reagent (Invitrogen) following the manufacturer's instructions and cultured for 10 days for positive selection. Small interfering RNAs (siRNAs) (GenePharma) were transfected into differentiated myotubes at a final concentration of 100 nM using Lipofectamine 3000.

For co‐culture studies, myotubes were washed with PBS and incubated with DMEM for 2 days. Conditioned media (CM) were collected and incubated with control IgG or anti‐FGF21 FL antibodies (#12180, Antibody and Immunoassay Services, HKU) according to the manufacture's instructions. For serum neutralization experiments, differentiated adipocytes were treated with DMEM supplied with collected mouse serum for 48 h before analysis.

### 2.10 Statistics

Data were expressed as mean ± SEM. Comparisons were performed via unpaired two‐tailed Student's t test, two‐way ANOVA followed by Bonferroni's post hoc test or analysis of covariance (ANCOVA) with the covariate of body weight. All *P* values less than 0.05 were considered statistically significant. Generally, * means *P* < .05, ** means *P* < .01 and *** means *P* < .001.

## 3 RESULTS

### 3.1 Overexpression of GLUT1, HK2 and PFKFB3 in mouse skeletal muscle increased glycolysis

**FIGURE 1:**
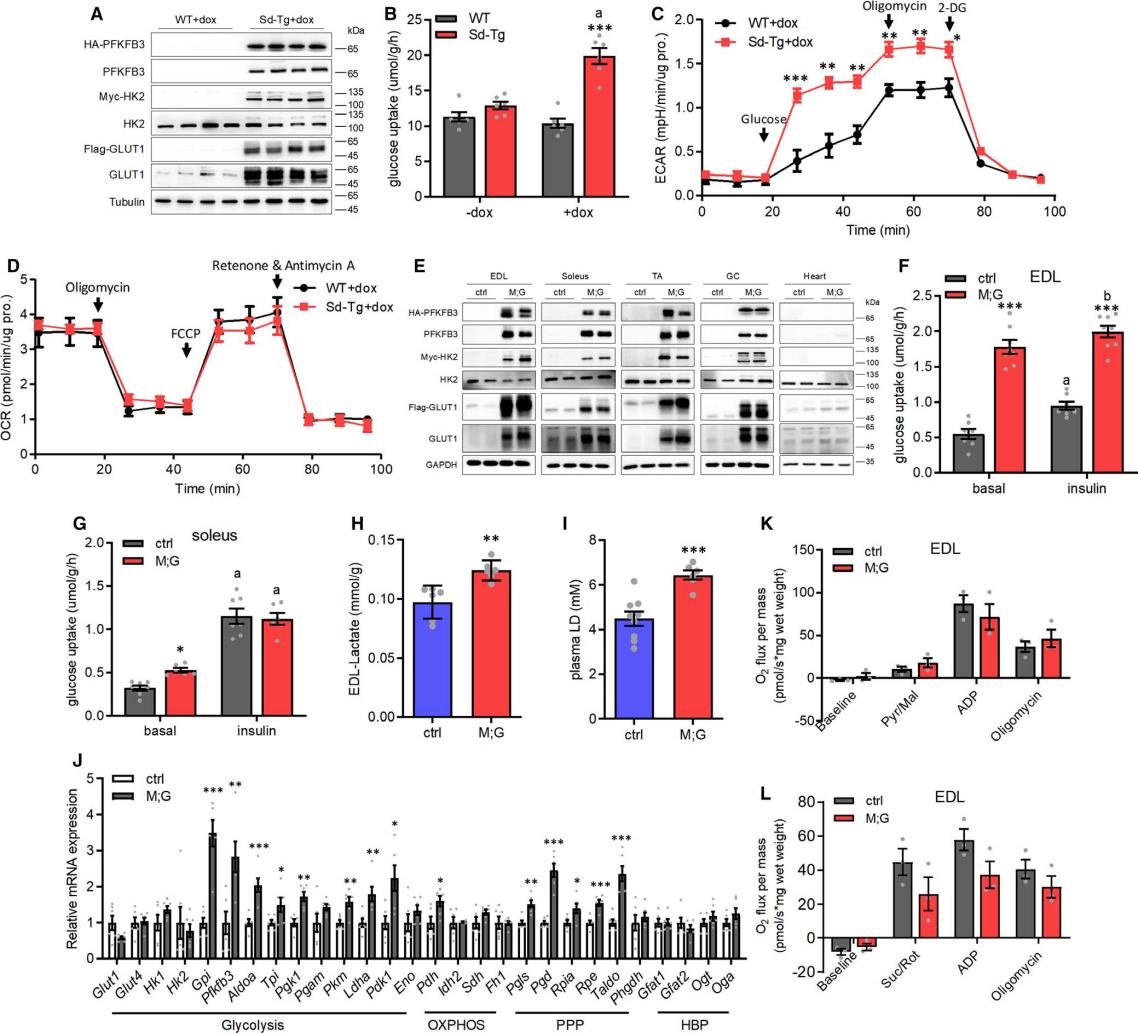
Enhanced glycolysis in cells with overexpression of *GLUT1*, *HK2* and *PFKFB3* genes. (A) Western blot analysis of MEFs under the treatment of doxycycline. (B) Glucose uptake in MEFs with or without doxycycline treatment. n = 6. Data were analysed via two‐way ANOVA with Bonferroni's post hoc test. (C) ECAR of MEFs under basal condition and followed by the sequential addition of glucose, oligomycin and 2‐DG under the treatment of doxycycline. n = 5. (D) OCR of MEFs under basal condition and followed by the sequential addition of oligomycin, FCCP, and rotenone and antimycin A under the treatment of doxycycline. n = 5. (E) Western blot analysis of indicated muscles from the male mice after 2 mo doxycycline‐treatment. (F) Glucose uptake in isolated EDL of the female mice at the age of 3‐4 mo. n = 7 control mice, n = 8 M;G mice. Data were analysed via two‐way ANOVA with Bonferroni's post hoc test. (G) Glucose uptake in isolated soleus of the female mice at the age of 3‐4 mo. n = 7 control mice, n = 6 M;G mice. Data were analysed via two‐way ANOVA with Bonferroni's post hoc test. (H) Lactate levels in EDL muscle of the mice. n = 6. (I) Plasma lactate levels in the mice at the age of 3 mo. n = 9 control mice, n = 6 M;G mice. (J) Relative mRNA expression of endogenous genes in TA muscle of the mice. n = 6. (K) Mitochondrial respiration rates of EDL muscle of the mice using pyruvate/malate (Pyr/Mal) as substrates. Pyr/Mal‐stimulated, ADP‐dependent and oligomycin‐induced respiration were shown. n = 3. (L) Mitochondrial respiration rates of EDL muscle of the mice using succinate/rotenone (Suc/Rot) as substrates. Suc/Rot‐stimulated, ADP‐dependent and oligomycin‐induced respiration were shown. n = 3. Values are means ± SEMs, *P < .05; **P < .01; ***P < .001 (unless stated, data were analysed via t test). For (B), (F) and (G), a indicates *P* < .001 and b indicates not significant (in the same genotype).

Based on previous reports^11^, ^12^, ^13^ and the critical role of PFKFB3 in promoting glycolytic flux, we designed a transgenic mouse model of co‐expressing human GLUT1 (an insulin‐independent glucose transporter), HK2 (the first rate‐limiting enzyme in glycolysis) and PFKFB3 (a critical glycolysis regulator), in a temporal‐ and spatial‐specific manner, by a polycistronic construct containing the 3 human encoding genes linked in tandem together with both rtTA/TRE and loxP‐STOP‐loxP (LSL) cassettes (Figure S1A). To obtain the transgenic mice, the linearized vector (pRosa‐CAG‐rtTA‐TRE‐LSL‐HA‐PFKFB3‐F2A‐Myc‐HK2‐P2A‐Flag‐GLUT1‐T2A‐EGFP‐WPRE) was recombined into a mouse Rosa26 BAC fragment for pronuclear micro‐injection. Three transgenic mouse lines (*Tg PFKFB3‐HK2‐GLUT1*) have been generated with two copies of the transgene in each line (Figure S1B‐D). The expression of the transgene has been further validated by Western blot performed in MEFs derived from the STOP‐deleted *Tg PFKFB3‐HK2‐GLUT1* mice (referred to as *Sd‐Tg*) under the treatment of doxycycline (Figure 1A). The *Sd‐Tg* MEFs exhibited increased levels of glucose uptake and ECAR, whereas the OCR did not change between the *Sd‐Tg* and control MEFs (Figure 1B‐D).

We next generated M;G mice by crossing *Tg PFKFB3‐HK2‐GLUT1* mice with *Muscle creatine kinase (Mck)‐cre* mice.^24^ Upon the treatment of doxycycline, the expression of the tag‐fused proteins was observed in various skeletal muscle types but not in cardiac muscle of M;G mice, or any muscles of their control cohorts (age‐matched *Mck‐cre* or *Tg PFKFB3‐HK2‐GLUT1* mice) (Figure 1E). 2‐Deoxy‐D‐glucose (2‐DG) uptake was significantly enhanced in EDL and soleus muscles under baseline conditions from M;G mice compared with controls (Figure 1F‐G). However, upon insulin stimulation, 2‐DG uptake was not increased in EDL muscle from the M;G mice while increased to a higher level in soleus muscle from the M;G mice, but remained comparable with the control mice (Figure 1F‐G). These results indicated an insulin‐independent enhancement of basal glucose uptake in muscles of M;G mice. Importantly, M;G mice exhibited higher levels of lactate in both muscle and plasma (Figure 1H‐I), indicating an elevation of glycolysis. In accordance, a number of genes involved in glycolytic and PPP pathways were up‐regulated in skeletal muscle of M;G mice (Figure 1J). Indeed, Oxygraph 2K analysis revealed that pyruvate and succinate driven mitochondrial oxidation in muscle were comparable between the control and M;G mice (Figure 1K‐L). Thus, we successfully generated a transgenic mouse model with enhanced glycolysis in muscle.

### 3.2 M;G mice exhibited tight glycaemia control and a lean phenotype

**FIGURE 2:**
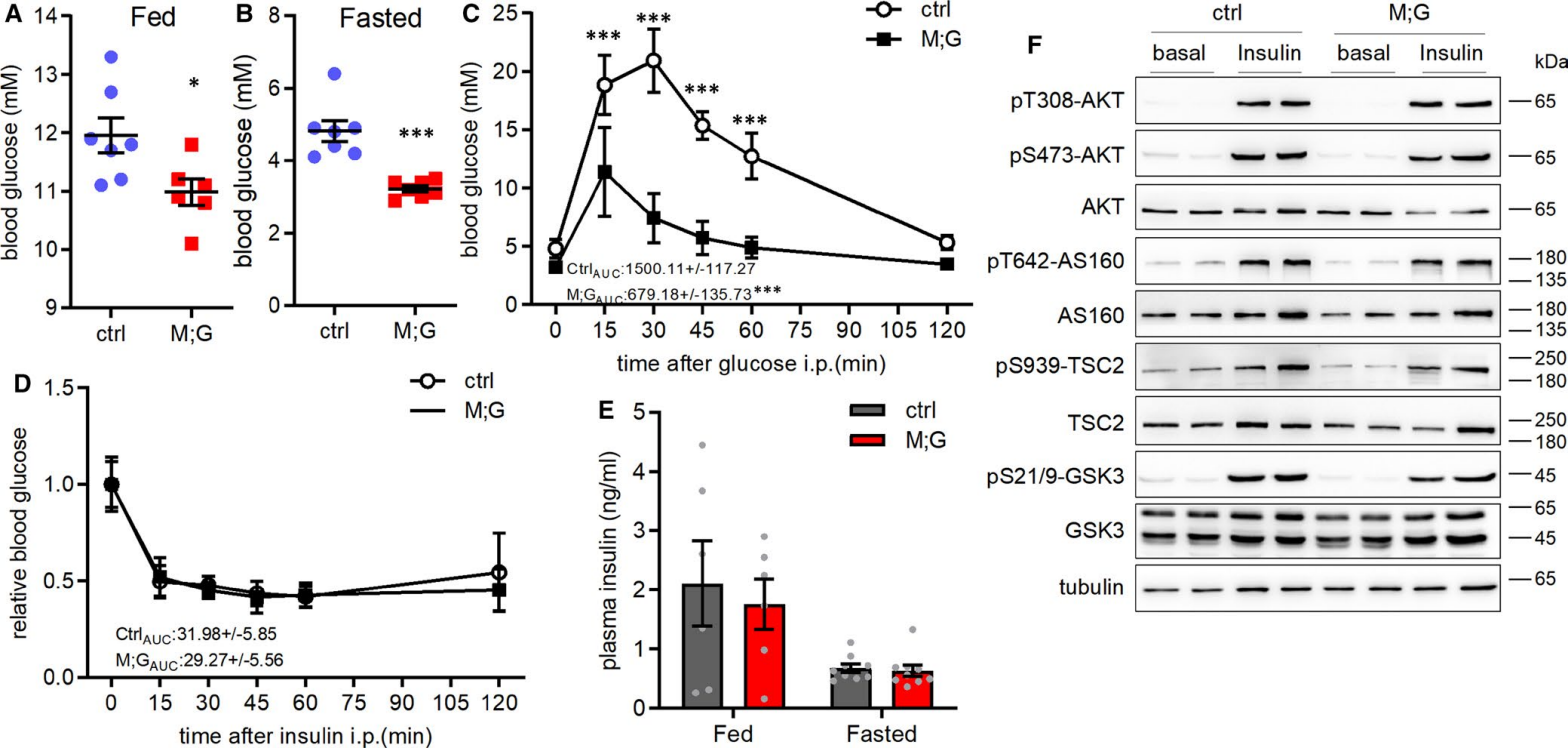
Improved insulin‐independent glucose disposal of the M;G mice. (A) Blood glucose in the random fed control and M;G mice at the age of 2‐3 mo. n = 7 control mice, n = 6 M;G mice. (B) Blood glucose in the overnight‐fasted control and M;G mice at the age of 2.5‐3.5 mo. n = 7. (C) IPGTT in control and M;G mice with 4‐week doxycycline treatment. Data were analysed via two‐way ANOVA with Bonferroni's post hoc test. The values showed the glucose area under the curve (AUC) during GTT. n = 7. (D) ITT in control and M;G mice at the age of 4.5 mo. Data were analysed via two‐way ANOVA with Bonferroni's post hoc test. The values showed the glucose under the curve during ITT. n = 7. (E) Plasma insulin levels of control and M;G mice. n = 6 fed mice, n = 9 fasted mice. (F) Phosphorylation and expression of the key components of insulin‐AKT pathway in soleus muscle of control and M;G mice in response to insulin. Values are means ± SEMs, *P < .05; **P < .01; ***P < .001 (unless stated, data were analysed via *t* test).

The M;G mice exhibited a normal, albeit lower blood glucose level compared with controls under ad libitum feeding condition, and marked hypoglycaemia under fasting condition, which was readily recovered after refeeding (Figure 2A‐B and Figure S2A). IPGTT demonstrated that M;G mice were much more tolerant to glucose challenge (Figure 2C). In addition, M;G and control mice displayed equivalent insulin‐stimulated glucose clearance and comparable plasma insulin levels (Figure 2D‐E). Notably, the M;G mice exhibited no enhancement in the systemic glucose tolerance in the absence of doxycycline‐treatment or 6 weeks after doxycycline withdrawal (Figure S2B‐C), indicating a transgene‐dependent effect. The insulin signalling pathway remained intact and undisturbed in muscle of the M;G mice at both baseline and in response to insulin stimulation (Figure 2F). Therefore, the M;G mice displayed a greater control of systemic glycaemia beyond that of the insulin signalling pathway.

**FIGURE 3:**
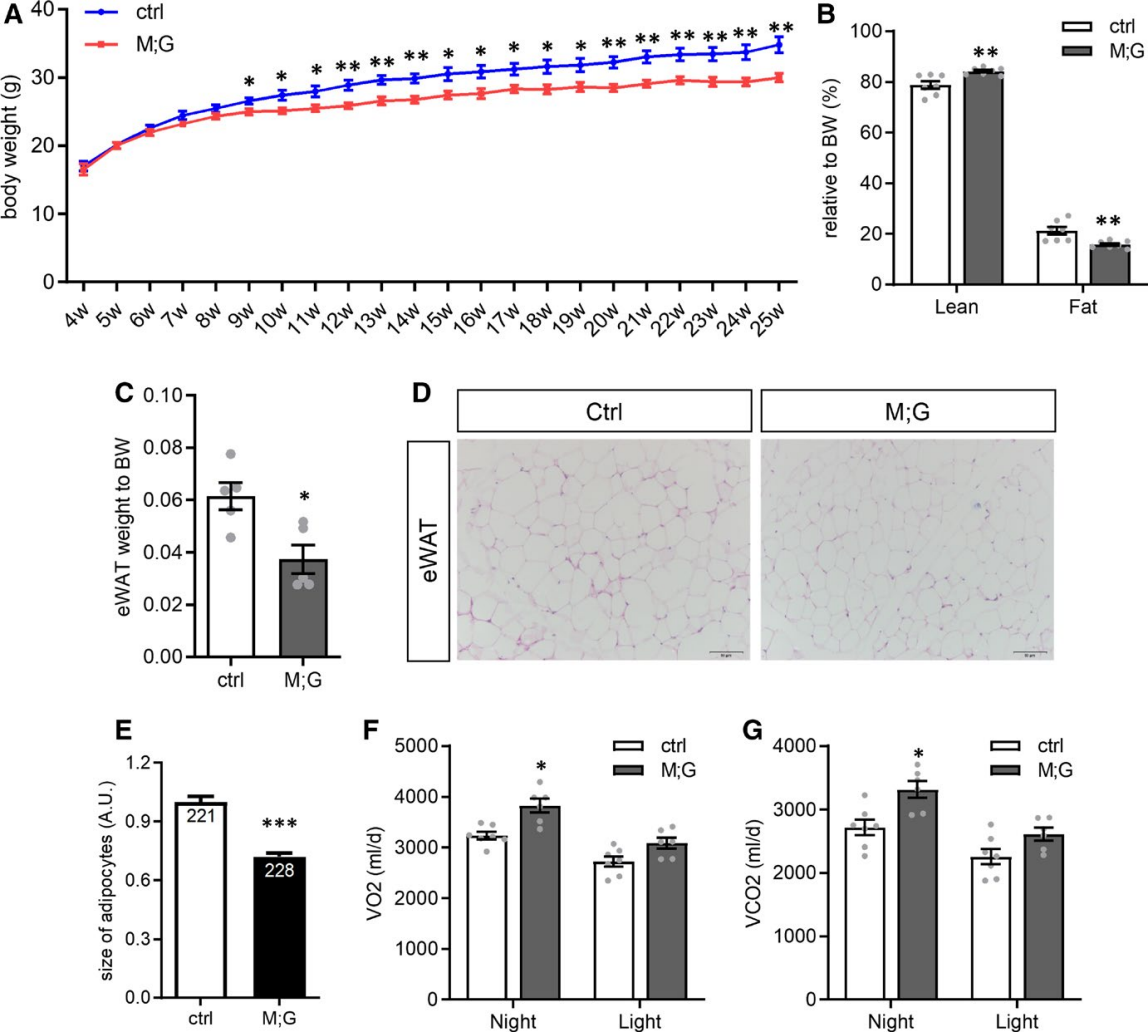
Reduced bodyweight gain of the M;G mice. (A) Growth curves of control and M;G mice from 4 to 25 wks of age. n = 7 control mice, n = 8 M;G mice. (B) Body composition of control and M;G mice measured at the age of 22 weeks. n = 7. (C) Relative eWAT weight of control and M;G mice at the age of 7‐8 mo. n = 5. (D) Histology of the adipose from control and M;G mice. Scale bar: 50 μm. (E) Cell size of the adipose from control and M;G mice. The numbers shown in the bar graphs indicated the number of cells measured in the experiments. (F) Oxygen consumption of the mice. n = 7 control mice, n = 6 M;G mice. (G) Carbon dioxide production of the mice. n = 7 control mice, n = 6 M;G mice. Data were analysed via ANCOVA in F‐G. Values are means ± SEMs, *P < .05; **P < .01; ***P < .001 (unless stated, data were analysed via *t* test).

Intriguingly, M;G mice exhibited a remarkable lower body weight compared with the controls one month after doxycycline treatment in spite of normal food intake (Figure 3A and Figure S3A). M;G mice also exhibited relatively increased lean mass and decreased fat mass as compared to the controls (Figure 3B). Consistent with decreased fat mass, smaller adipocytes were found in the adipose tissue of M;G mice (Figure 3C‐E). There were no abnormalities in tissue weight and histology in liver of the M;G mice (Figure S3B‐C). We performed indirect calorimetric analysis and found that M;G mice exhibited significant increases in oxygen consumption (VO_2_) and carbon dioxide production (VCO_2_) during the night (Figure 3F‐G). While the ANCOVA‐adjust energy expenditure (EE) was similar in the two genotypes after the effects of mass were eliminated, the EE adjusted for body weight was significantly higher in M;G than in control mice during the night (Figure S3D‐E). Moreover, M;G mice showed a moderate increased respiratory exchange ratio (RER) and elevated locomotor activity at night (Figure S3F‐H). In addition, the running ability of M;G mice was comparable with control mice (Figure S3I). The peak grip forces of forelimb and four limbs also did not change between the M;G and control mice (Figure S3J‐K). Therefore, the function of the skeletal muscle in the M;G mice was normal. Furthermore, no difference was found in the physiological index of M;G and control mice without the treatment of doxycycline (Figure S3L). Collectively, the mice with skeletal muscle‐specific overexpression of GLUT1, HK2 and PFKFB3 exhibited a leaner phenotype with reduced fat mass, increased oxygen consumption and carbon dioxide production.

### 3.3 M;G mice exhibited reduced capacity for lipid uptake and oxidation in the skeletal muscle

**FIGURE 4:**
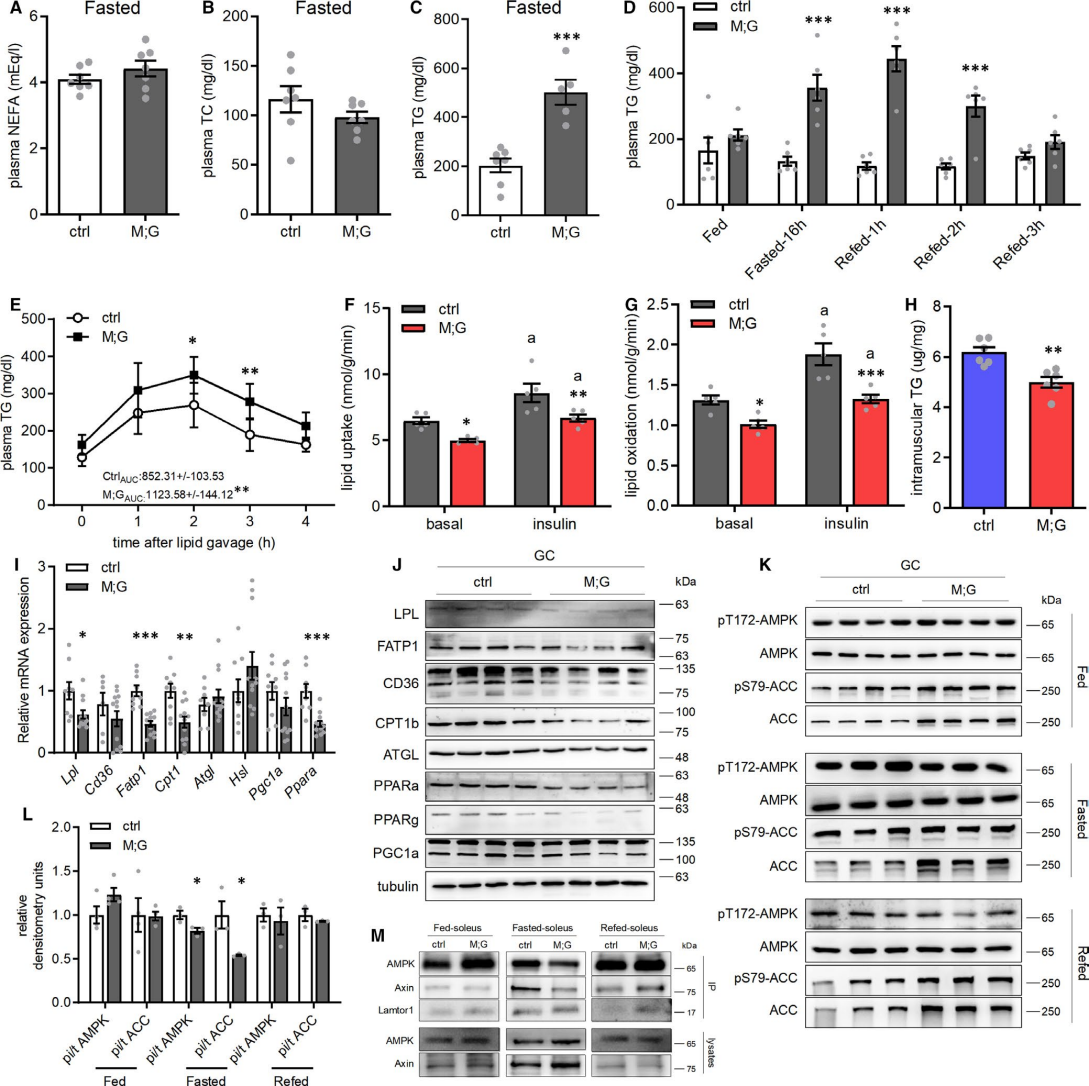
Reduced capacity of lipid uptake and oxidation in the muscle of the M;G mice. (A‐C) Plasma NEFA (A), TC (B) and TG (C) levels in the overnight‐fasted mice at 3‐4 mo of age. n = 7. (D) Plasma TG levels in the fed, 16h‐fasted and fasting‐refed control and M;G mice. n = 6. (E) Plasma TG levels after lipid oral gavage in control and M;G mice at the age of 7 mo. Data were analysed via two‐way ANOVA with Bonferroni's post hoc test. The values showed the TG area under the curve during lipid tolerance test. n = 7 control mice, n = 6 M;G mice. (F) Lipid uptake in isolated soleus muscle of the 4‐month‐old female mice. n = 5. Data were analysed via two‐way ANOVA with Bonferroni's post hoc test. (G) Lipid oxidation in isolated soleus muscle of the 4‐month‐old female mice. n = 5. Data were analysed via two‐way ANOVA with Bonferroni's post hoc test. (H) TG levels in TA muscle from the fasted mice at the age of 4 months. n = 6. (I) mRNA expression of lipid metabolic genes in GC muscle from the overnight‐fasted mice. n = 9 control mice, n = 12 M;G mice. (J) Protein expression of lipid metabolic genes in GC muscle from the overnight‐fasted mice. (K) Expression and phosphorylation of AMPK and ACC in GC muscle from the fed, fasted and fasting‐refed mice. (L) Quantification of the pi‐AMPK/AMPK, pi‐ACC/ACC ratios in (K). (M) Representative Western blots showing the amount of AMPK and Axin for soleus muscle from the mice under different feeding states. Lamtor1 was immunoprecipitated from tissue extracts and then immunoblotted with AMPK, Axin and Lamtor1 antibody to determine the amount of each protein. Unless stated, male mice were applied for the experiments. Values are means ± SEMs, *P < .05; **P < .01; ***P < .001 (unless stated, data were analysed via *t* test). For (F) and (G), a indicates *P* < .05 (insulin vs basal of the same genotype)

It is largely believed that the balance of glucose and lipid metabolism plays an essential role in sustaining metabolic homeostasis. To study the influence of the enhanced glycolysis in skeletal muscle on whole‐body lipid metabolism, we examined the lipid profile in M;G and control mice. During the fed state, both M;G and control mice showed similar levels of NEFA, TC and TG in their plasma. However, at the fasting state, M;G mice had elevated levels of TG but similar levels of NEFA and TC in the plasma (Figure S4A‐C and Figure 4A‐C). The elevated level of TG during fasting in M;G mice returned back to normal within 3 hours after refeeding (Figure 4D). Therefore, the M;G mice exhibited hyperlipidaemia during adaption to starvation but managed to maintain lipid homeostasis during the fasting‐refeeding cycle. When challenged with an oral lipid load, the M;G mice exhibited comparable plasma NEFA level but higher plasma TG level relative to controls (Figure S4D and Figure 4E), indicating a reduced capacity for blood TG clearance. In response to insulin stimulation, the basal and insulin‐stimulated lipid uptake and oxidation were all significantly lower in the M;G mice compared with the controls (Figure 4F‐G). In accordance, there was a decrease of ~20% intramuscular TG content in the M;G mice (Figure 4H)

Moreover, genes involved in the FA transport and oxidation processes were remarkably decreased in the skeletal muscle from overnight‐fasted M;G mice, in both RNA and protein levels (Figure 4I‐J). To further investigate lipid oxidation, we assessed the activity of AMPK by detecting AMPKα Thr172 phosphorylation and ACC phosphorylation in the muscles of mice. AMPK activity in the fed and refed states was comparable between the M;G and control mice, but its activity was significantly lower under fasted state when compared to the control mice (Figure 4K‐L). In addition, protein co‐immunoprecipitation analysis showed decreased recruitment of AMPK and Axin to a lysosome‐anchored metabolite sensing complex^25^ in muscles of the M;G mice (Figure 4M), suggesting the impaired lipid utilization in the skeletal muscle of the M;G mice may involve a metabolite sensing mechanism.

### 3.4 M;G mice were alleviated from diet‐induced obesity and insulin resistance with increased energy expenditure in the adipose tissue

**FIGURE 5:**
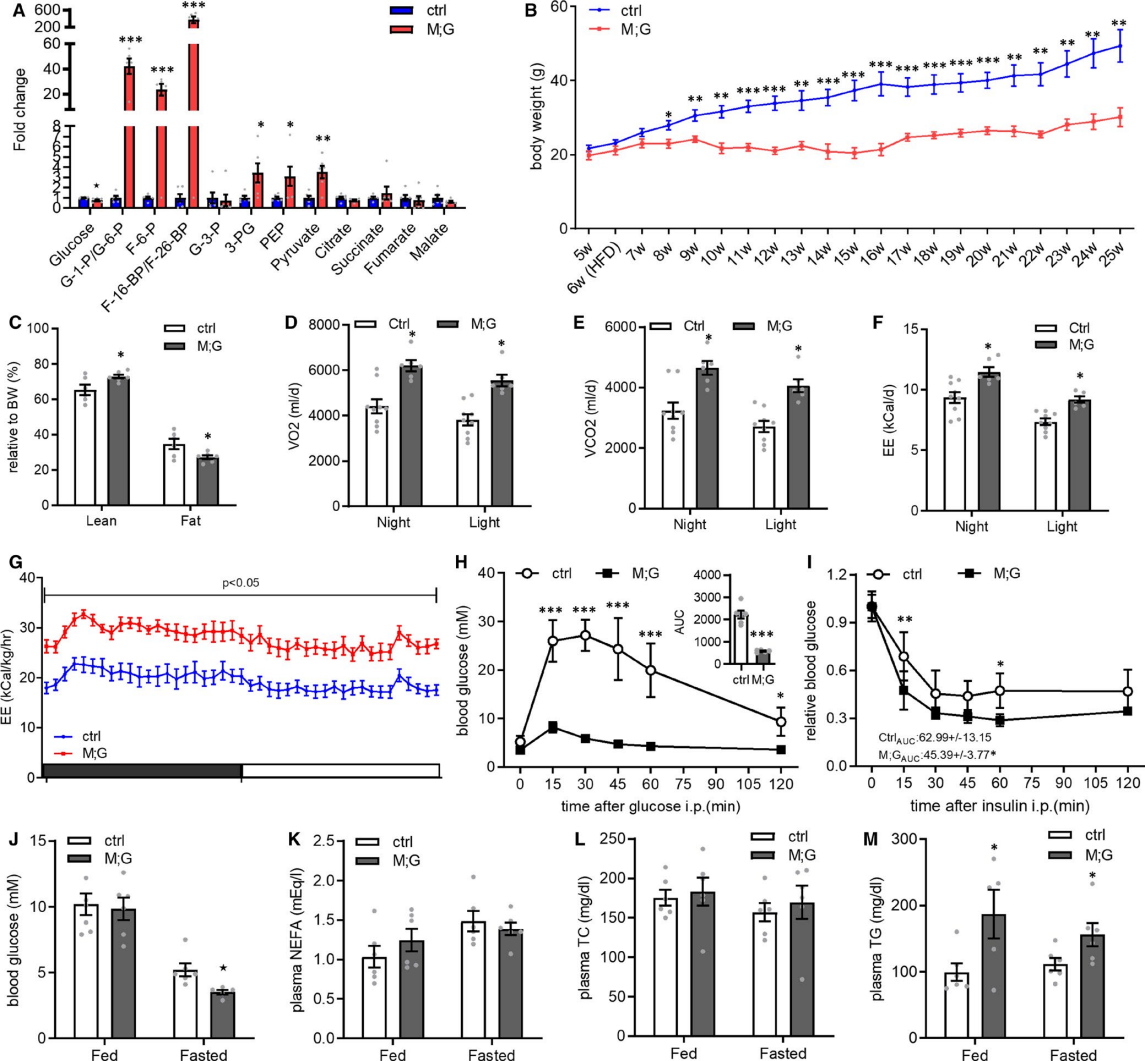
Alleviation of HFD‐induced obesity and insulin resistance in the M;G mice. (A) Fold changes of glucose metabolic intermediates in GC muscle of the mice fed on HFD. n = 6. (B) Growth curves of the HFD‐fed control and M;G mice from 5 to 25 wks of age. n = 6. (C) Body composition of the HFD‐fed mice at the age of 7‐8 mo. n = 6. (D) Oxygen consumption of the mice fed on HFD. n = 9 control mice, n = 6 M;G mice. (E) Carbon dioxide production of the mice fed on HFD. n = 9 control mice, n = 6 M;G mice. (F) EE in control and M;G mice fed on HFD. n = 9 control mice, n = 6 M;G mice. Data were analysed via ANCOVA in D‐F. (G) Adjust energy expenditure per hour during the light/dark cycle of the mice fed on HFD. The measurements were normalized to mouse bodyweight. n = 9 control mice, n = 6 M;G mice. (H) IPGTT in the HFD‐fed control and M;G mice at the age of 4.5 mo. Insert: the glucose area under the curve during IPGTT. n = 6 control mice, n = 5 M;G mice. (I) ITT in the HFD‐fed control and M;G mice at the age of 4‐4.5 mo. The values showed the glucose area under the curve during ITT. n = 6. Data were analysed via two‐way ANOVA with Bonferroni's post hoc test in H‐I. (J‐M) Blood glucose (J), plasma NEFA (K), TC (L) and TG (M) levels in the HFD‐fed mice at the age of 4‐4.5 mo. n = 6. Values are means ± SEMs, *P < .05; **P < .01; ***P < .001 (unless stated, data were analysed via t test). G‐6‐P/G‐1‐P: glucose‐monophosphate, F‐6‐P: fructose‐6‐phosphate, F‐1,6‐BP/F‐2,6‐BP: fructose‐bisphosphate, 3‐PG: 3‐phosphoglycerate, PEP: phosphoenolpyruvic acid.

HFDs are well known to induce insulin resistance and impair glucose and lipid homeostasis. Similar to the chow‐fed conditions, the HFD‐fed M;G mice exhibited up‐regulation of glucose metabolic‐related genes and down‐regulation of genes and proteins involved in lipid oxidation in the skeletal muscle (Figure S5A‐C). Then, we compared the profile of glucose metabolites in skeletal muscles of the HFD‐fed M;G and control mice. Consistent with a highly glycolytic state, the skeletal muscle of the HFD‐fed M;G mice contained markedly elevated levels of glycolytic metabolic intermediates (Figure 5A). The enhanced glycolysis was accompanied by a drop of free glucose (Figure 5A), implying that the high amount of glucose absorbed has been rapidly mobilized. Meanwhile, the intermediate metabolites in TCA cycle were not altered (Figure 5A). These results further verified the highly glycolytic metabolic profile in the skeletal muscles of the M;G mice.

Under HFD conditions, the M;G mice weighted significantly less than their age‐matched controls while with similar food intakes (Figure 5B and Figure S5D). Body composition analysis revealed decreased fat mass in the HFD‐fed M;G mice (Figure 5C). While no difference was observed in locomotor activity, a moderately elevated RER and marked increases in VO2, VCO2 were found in the HFD‐fed M;G mice based on indirect calorimetry (Figure S5E‐G and Figure 5D‐E). Moreover, HFD‐fed M;G mice exhibited higher adjusted EE and ANCOVA‐adjusted EE than control mice during both the night and daytime (Figure 5F‐G). Therefore, the lean phenotype of the HFD‐fed M;G mice was accompanied with an increased systemic energy expenditure.

We next investigated the metabolic profiles in mice under HFD. HFD‐fed M;G mice were more tolerant to glucose challenge and more sensitive to insulin stimulation compared with the HFD‐fed control littermates (Figure 5H‐I). HFD‐fed M;G mice also exhibited normal blood glucose, plasma NEFA, TC and fasting plasma NEFA and TC levels, but a lower fasting blood glucose level (Figure 5J‐L). Notably, the plasma levels of TG were elevated in the M;G mice under both feeding and fasting conditions (Figure 5M). The TG content and fatty liver phenotype did not change between the HFD‐fed M;G and control mice (Figure S5H‐I). Taken together, the fact which M;G mice were largely protected against HFD‐induced obesity and insulin resistance, emphasized that the enhanced glycolysis in skeletal muscle could induce beneficial systemic effects even under an over‐nutritional condition.

**FIGURE 6:**
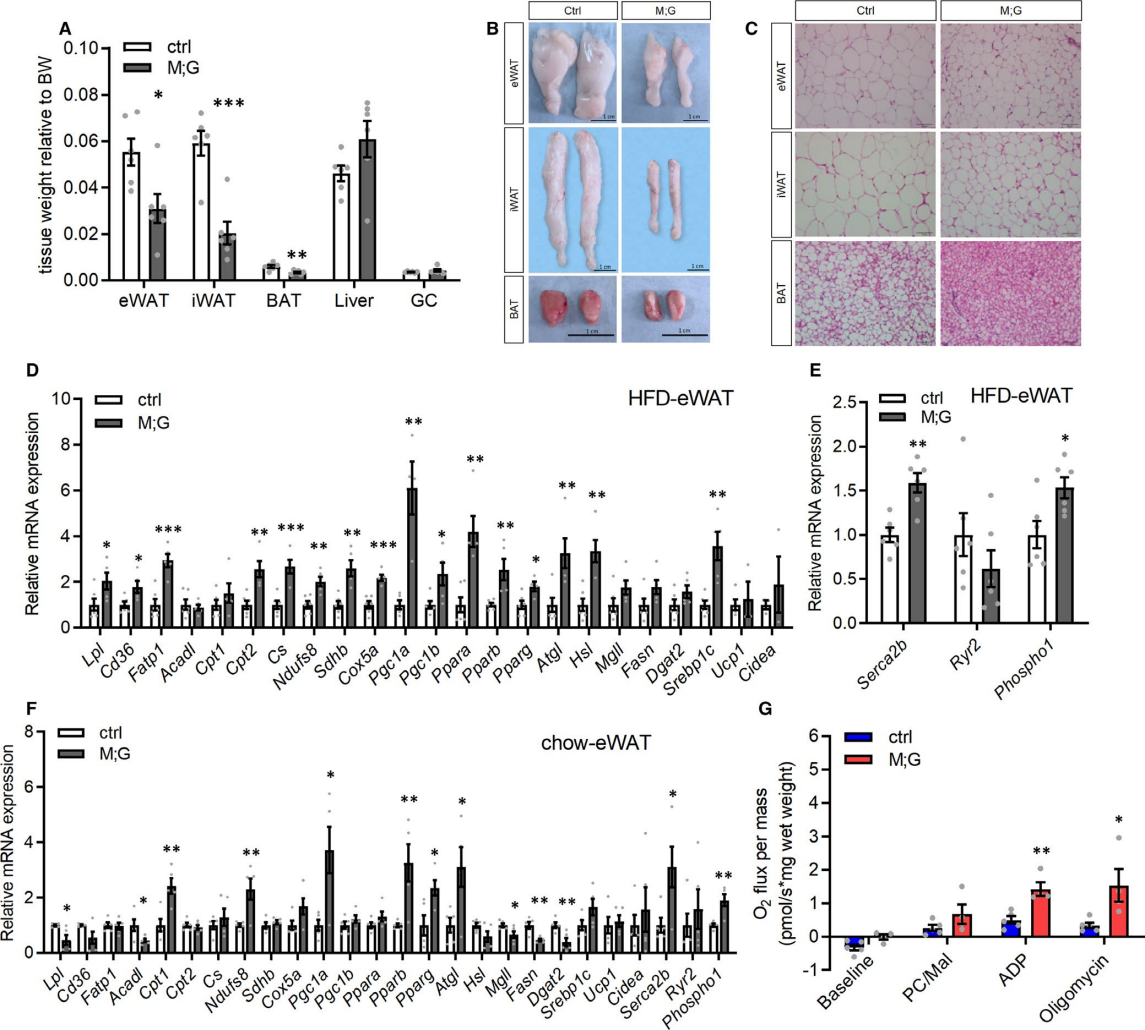
Up‐regulation of lipid metabolism and thermogenesis in visceral white adipose tissue of the M;G mice. (A) Relative tissue weight of the HFD‐fed mice at the age of 7‐8 mo. n = 6. (B) Photographs of eWAT, iWAT and BAT from control and M;G mice fed on HFD. Scale bar: 1 cm. (C) Histology of adipose from the HFD‐fed control and M;G mice. Scale bar: 50 μm. (D‐E) mRNA expression of indicated genes in eWAT from the HFD‐fed control and M;G mice. n = 6 control mice, n = 5 M;G mice. (F) mRNA expression of indicated genes in eWAT from the chow diet‐fed control and M;G mice. n = 5. (G) Mitochondrial respiration rates of eWAT of the chow diet‐fed mice using palmitoylcarnitine/malate (PC/Mal) as substrates. PC/Mal‐stimulated, ADP‐dependent and oligomycin‐induced respiration were shown. n = 5 control mice, n = 4 M;G mice. Values are means ± SEMs, *P < .05; **P < .01; ***P < .001 (*t* test).

In agreement with the decreased body fat composition (Figure 5C), visceral white adipose tissue (eWAT), subcutaneous white adipose tissue (iWAT) and brown adipose tissue (BAT) were all smaller in the HFD‐fed M;G mice relative to their controls (Figure 6A‐B). Histologic analysis also revealed smaller adipocytes in the M;G mice (Figure 6C). The eWAT of the HFD‐fed M;G mice exhibited dramatically elevated expression of key genes involved in lipid metabolism (Figure 6D). The effects were less pronounced in iWAT and BAT of the HFD‐fed M;G mice (Figure S6A‐B). The expression of the canonical thermogenic genes, *Ucp1* and *Cidea,* was not altered in all three types of adipose tissue (Figure 6D and Figure S6A‐B), whereas higher expression of *sarco/endoplasmic reticulum Ca* ^2^ *^+^‐ATPase 2b* (*Serca2b*) and *phosphatase orphan 1* (*Phospho1*), the UCP1‐independent regulators of fat thermogenesis,^26^, ^27^ were observed in eWAT of the HFD‐fed M;G mice (Figure 6E). Moreover, the expression of lipid metabolic genes as well as *Serca2b* and *Phospho1* was also increased in eWAT but not in iWAT of the chow diet‐fed M;G mice (Figure 6F and Figure S6C). Consistent with an enhanced lipid oxidation in the adipose tissue, there were significant increases in palmitoylcarnitine (PC)/malate‐driven state 3 (maximal ADP‐stimulated) and state 4 (uncoupled) mitochondrial respiration rates in eWAT of the chow diet‐fed M;G mice (Figure 6G). Thus, the enhanced lipid metabolism and thermogenesis in eWAT corroborated the elevated energy expenditure and alleviation of obesity found in the M;G mice.

### 3.5 Muscle FGF21 derived in a ChREBP‐dependent manner possibly contributed to the favourable metabolic phenotype in M;G mice

**FIGURE 7:**
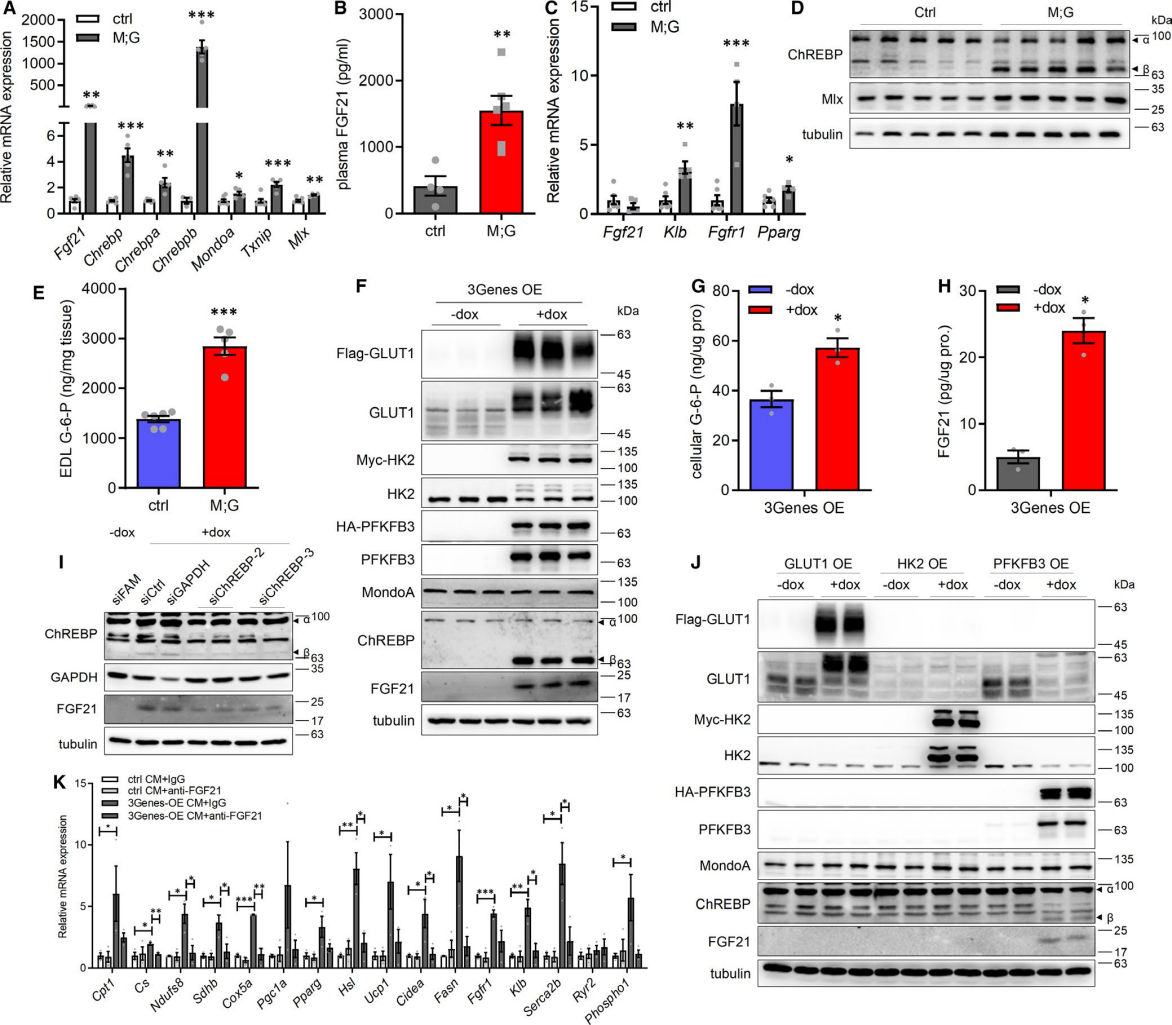
Enhanced glycolysis in muscle regulated systemic homeostasis through endocrine actions of FGF21. (A) mRNA expression of indicated genes in the M;G and control mice fed on HFD. n = 6 control mice, n = 5 M;G mice. (B) Plasma FGF21 levels in the mice fed on HFD for 5 months. n = 4 control mice, n = 6 M;G mice. (C) mRNA expression of indicated genes in eWAT of the HFD‐fed mice. n = 6 control mice, n = 5 M;G mice. (D) Western blot analysis of ChREBP and Mlx in GC muscle from the mice fed on HFD. (E) G‐6‐P levels in EDL muscle of the HFD‐fed mice. n = 6. (F) Western blot analysis of the 3Genes‐OE C2C12 myotubes. (G) Cellular G‐6‐P levels in the 3Genes‐OE myotubes under the treatment of doxycycline. n = 3. (H) FGF21 levels in CM from the indicated myotubes. n = 3. (I) Western blot analysis of the 3Genes‐OE myotubes transfected with either ChREBP‐specific or control siRNA, with or without the treatment of doxycycline. FAM‐control indicates fluorescein amidites‐labelled control siRNA. (J) Western blot analysis of the indicated C2C12 myotubes under the treatment of doxycycline. (K) mRNA expression of indicated genes in the primary visceral white adipocytes treated with either control or 3Genes‐OE CM, with or without the treatment of anti‐FGF21 antibody. n = 3. Values are means ± SEMs, *P < .05; **P < .01; ***P < .001 (*t* test).

To meet high aerobic catabolic demands for ATP, contracting muscle synthesizes and secretes cytokines and other peptides to act both locally and distally to regulate metabolism and adjust systemic energy balance.^28^, ^29^ We thus suggested that the enhanced glycolysis in skeletal muscle of M;G mice regulates whole‐body energy homeostasis through the induction of myokines. We firstly examined the expression of myokines which are responsible for modulating systemic energy homeostasis by Quantitative RT‐PCR and found that *Fgf21* was the most up‐regulated gene in the muscle of M;G mice fed on both chow diet and HFD (Figure S7A and Figure 7A). Moreover, the levels of FGF21 in plasma were also remarkably elevated under both diet conditions in M;G mice (Figure S7B and Figure 7B). FGF21 has been shown to promote oxidative metabolism in the adipose tissue.^30^ However, *Fgf21* mRNA expression was not altered in eWAT, iWAT, BAT and liver between the HFD‐fed M;G and control mice (Figure 7C and Figure S7C‐E), suggesting that only skeletal muscle was responsible for the increased FGF21 secretion in the M;G mice. Accordingly, the expression of *Fgfr1*, *Klb* and *Ppar*, which govern the metabolic actions of FGF21 in adipose tissue,^31^, ^32^, ^33^ was significantly increased in eWAT in the M;G mice under both diet conditions, but not in iWAT, BAT or liver of the HFD‐fed M;G mice (Figure 7C and Figure S7C‐F), supporting the notion that eWAT was the target tissue of muscle‐derived FGF21 in this context. Furthermore, treatment with serum from M;G mice induced the increased expression of many lipid metabolic and thermogenic genes in primary visceral adipocytes, while blocking FGF21 in the same serum suppressed the induction (Figure S7G), suggesting that FGF21 may play an important role in the regulation of lipid metabolism.

Next, we explored the regulation for the increased expression of FGF21 in the skeletal muscle of the M;G mice. Consistent with previous findings, PPARα and PPARγ were down‐regulated with the attenuated lipid metabolism in muscles of the M;G mice (Figure 4J and Figure S5C). Stress‐associated eukaryotic initiation factor 2α (eIF2α)‐ATF4 pathways were not altered either (Figure S7H). However, the expression of ChREBPβ, a shorter and constitutively active isoform of the ChREBP, was dramatically elevated in the skeletal muscles of the HFD‐fed M;G mice, whereas its paralogue *MondoA*, their functional obligatory partner Mlx and their target gene *Txnip* were only modestly increased (Figure 7A and 7D). The enhanced expression of ChREBPβ was also observed in the skeletal muscle of the M;G mice fed on chow diet (Figure S7I‐J). The level of G‐6‐P, a glucose metabolite known to activate ChREBP/MondoA, was elevated as well in EDL muscle of the M;G mice regardless of diets (Figure 7E and Figure S7K).

To substantiate the FGF21 regulation by ChREBP in the skeletal muscles of the M;G mice and examine its functional impact, we recapitulated the enhanced glycolysis in C2C12 myotubes overexpressing PFKFB3, HK2 and GLUT1 (3Genes‐OE). These cells showed increased expression of ChREBPβ and FGF21, elevated cellular G‐6‐P concentration and enhanced FGF21 secretion (Figure 7F‐H). Knockdown of ATF4 did not impair the activation of ChREBP and FGF21 in the 3Genes‐OE myotubes (Figure S7L). Knockdown of ChREBP blunted the induction of FGF21, whereas knockdown of MondoA did not (Figure 7I and Figure S7 M). Interestingly, the overexpression of PFKFB3 alone in C2C12 myotubes moderately up‐regulated the expression of ChREBPβ and FGF21, whereas the overexpression of either HK2 or GLUT1 did not (Figure 7J). Thus, enhanced glycolysis, which may critically dependent on PFKFB3, stimulated ChREBP to promote the expression of FGF21 in the skeletal myocytes. The incubation of CM from the 3Genes‐OE myotubes led to increased expression of many lipid metabolic and thermogenic genes in primary visceral adipocytes (Figure 7K). Furthermore, FGF21 blocking antibody blunted the induction of lipid metabolic genes by 3Genes‐OE myotubes‐CM (Figure 7K), strongly suggesting that FGF21 derived from the myotubes directly promoted the metabolic and thermogenic reprogramming in WAT.

In summary, enhanced glycolysis activated ChREBP in skeletal myocytes, which stimulated FGF21 production to elevate lipid metabolism and energy expenditure in adipocytes. Thus, under a highly glycolytic state, skeletal myocytes could engage in direct glucose‐sensing pathway to regulate systemic homeostasis.

## 4 DISCUSSION

Altogether, our study shows that elevating glycolytic metabolism in skeletal muscle could combat diet‐induced IR and obesity through its profound effects on systemic metabolism and adipose tissue (Figure S8). The enhanced anaerobic glycolytic metabolism and metabolites in muscle of M;G mice may have engaged in two inter‐connected glucose‐sensing pathways to remodel the metabolic network: reducing AMPK activity, which led to attenuated fatty acid oxidation in muscle, and activating ChREBP/FGF21 axis, which resulted in elevated lipid metabolism and thermogenesis in eWAT. Thus, the enhanced glucose metabolism in skeletal muscle could be intrinsically balanced by an increase in lipid metabolism in adipose tissue to maintain systemic metabolic homeostasis under nutrient‐replete conditions.

Regulation of cellular glycolytic metabolic flux involves the coordination of cell signalling, enzyme activities and levels of metabolites. PFKFB3 is the rate‐limiting enzyme responsible for commitment towards glycolysis through the production of F‐2,6‐BP. In our study, we identified PFKFB3 was the main glycolytic enzyme responsible for sensing glucose in skeletal myocytes, further contributing to systemic metabolic benefit. Therefore, as PFKFB3 protein is unstable in skeletal muscle,^34^ tissue‐specific targeting of PFKFB3 either through protein expression level, protein stability or enzyme activity may represent novel therapeutics for the treatment of metabolic disorders related to obesity. Remarkably, the M;G mice remained highly sensitive to glucose and insulin irrespective of diets. In sharp contrast, the GLUT1‐overexpressing mice have elevated glucose flux via the hexosamine biosynthetic pathway (HBP) in muscle,^35^ which is associated with enhanced O‐GlcNAcylation of the insulin signalling pathway and insulin resistance.^36^, ^37^ It is plausible that the enhanced glycolytic flux, including a high conversion rate of F‐6‐P to F‐1,6‐BP mediated by PFK1, diverted F‐6‐P away from the HBP and protected the insulin signalling components from inhibition in the M;G mice. Thus, our study provides novel clues for glycolytic flux regulation and manipulation.

Glucose and its derived metabolites are sensed in various types of cells and tissues, including the hypothalamus, pancreatic islets, liver, adipose tissue and skeletal myocytes.^38^, ^39^, ^40^, ^41^ The central mediators of glucose sensing are ChREBP and its paralog MondoA. ChREBP is a transcription factor highly expressed in liver and adipose tissue, with moderate expression in kidney, skeletal muscle and intestine.^42^ The transcriptional activity of ChREBP is activated by glucose metabolites (G‐6‐P, F‐2,6‐BP, Xu‐5‐P), acetylation or O‐GlcNAcylation.^43^ ChREBP, especially the constitutively active ChREBPβ isoform, can be significantly induced in liver with a high carbohydrate diet^44^, ^45^ and promoted the expression of lipogenic genes (eg Acc, Fasn, Scd1 and Elovl6) and Fgf21.^46^, ^47^ On the other hand, MondoA has been shown to be activated by intracellular glycolytic metabolites during glucose stimulation in skeletal muscle cells to regulate the expression of glycolytic and lipogenic genes.^48^, ^49^ Under chronic caloric excess, MondoA promoted lipid storage and insulin resistance via up‐regulation of lipogenic genes and Txnip.^50^, ^51^ The enhanced glucose metabolism and elevated glycolytic metabolites in the skeletal muscle of M;G mice profoundly stimulated the expression of ChREBP, especially its active form ChREBPβ together with FGF21, while only modestly increased the levels of *MondoA* and *Txnip*. In addition, ChREBP, but not MondoA, was responsible for FGF21 induction in cultured myocytes. Thus, under the context of a high glycolytic state, the ChREBPβ/FGF21 axis played a dominant role in skeletal muscle as the glucose sensor in promoting systemic metabolic homeostasis. Although current studies do not clearly distinguish the regulatory mechanisms of ChREBP and MondoA, our study uncovered that ChREBP and MondoA regulate glucose sensing in different manners, raising critical questions of the key determinants in glycolysis and metabolites mediating the effects.

The fact that the expression of PFKFB3 alone in myocytes could promote a moderate FGF21 expression suggested the importance of high levels of F‐2,6‐BP or PFK activity not only in glycolysis, but also in glucose sensing. In both mice and healthy humans, acute endurance exercise is able to elevate serum level of FGF21 for systemic metabolic regulation, although the contributing organs and underlying mechanisms for its induction remain incompletely understood.^52^, ^53^, ^54^ Our results implied that enhanced glycolysis in muscle may also be able to stimulate FGF21 production. In addition, recent reports demonstrated that high carbohydrate ingestion was also accompanied by FGF21 induction in human leg muscles.^55^ Whether ChREBP is involved in these processes warrants further investigation. Nonetheless, this study highlights a glucose‐sensing pathway in skeletal muscles that could be manipulated to promote systemic metabolic benefit. Notably, this pathway was independent of the recently described ATF4‐mediated FGF21 induction in the presence of either ER stress or mitochondria dysfunction in skeletal muscles.^56^, ^57^, ^58^

Interest in FGF21 exploded with the discovery of its potent metabolic regulating effects. The systemic beneficial effects of FGF21 is mainly ascribed to the UCP1‐dependent browning of white adipose tissue.^30^, ^57^, ^59^ However, the effects of FGF21 may not be limited to the adipose browning.^60^ In eWAT of the M;G mice, the greatly enhanced oxidative metabolism and lipid turnover, together with the induction of Serca2b and Phospho1, strongly suggested a UCP1‐independent thermogenesis mechanism for the increased energy expenditure mediated by FGF21. Furthermore, it is reported that FGF21 might also increase futile cycle of lipogenesis and lipolysis in WAT,^61^ which is consistent with the expression of lipid metabolic genes found in the adipose tissue of the M;G mice. As AAV‐FGF21 gene therapy have resulted in marked reductions in bodyweight and insulin resistance in HFD feeding and ob/ob mice,^62^ FGF21 can be a potential target to treat obesity and insulin resistance. While the mechanistic base for the difference between eWAT and iWAT in the FGF21 response is currently unknown, the uniformed reductions of adipose tissues in the M;G mice might involve a systemic effect of lipid utilization and distribution.

Previous studies showed that enhanced glucose intake in transgenic mice expressing human GLUT4 under its own promoter improved insulin sensitivity, but reduced lipid oxidation in the skeletal muscle and did not alleviate obesity under HFD.^11^ In the skeletal muscles of M;G mice, we identified a decreased recruitment of AMPK into the glucose‐sensing v‐ATPase‐Ragulator‐AXIN/LKB1‐AMPK protein complex reported recently,^25^ leading to decreased phosphorylation of AMPK, which results in less muscle lipid oxidation and subsequent fasting hyperlipidaemia under fasting condition. However, the M;G mice still managed to maintain a relatively normal lipid homeostasis under chow diet possibly as a result of the enhanced lipid oxidation in the remote adipose tissue, resulting in a fundamental difference between the M;G mice and the Glut4 transgenic mice.

In conclusion, our study uncovered that increased insulin‐independent glycolysis flux in the skeletal muscle could remodel the whole‐body metabolic network and improve the physical well‐being of mice, especially under HFD. The coordination between enhanced glycolysis in skeletal muscle and lipid metabolism in white adipose tissue suggested an intrinsic homeostatic mechanism regarding glucose/lipid balance. Further understanding of glycolysis, especially the glucose‐sensing pathways in skeletal muscle will be beneficial for the treatment of obesity, diabetes and associated metabolic disorders.

## CONFLICT OF INTEREST

All authors declare no conflict of interest.

## AUTHOR CONTRIBUTION

**Cong Xiang:** Conceptualization (lead); Data curation (lead); Formal analysis (lead); Investigation (lead); Software (lead); Validation (equal); Writing‐original draft (lead); Writing‐review & editing (lead). **Yannan Zhang:** Data curation (equal); Formal analysis (supporting); Investigation (equal); Software (supporting); Writing‐review & editing (supporting). **Qiaoli Chen:** Data curation (supporting); Investigation (supporting); Methodology (supporting); Writing‐review & editing (supporting). **Aina Sun:** Data curation (supporting); Methodology (supporting); Writing‐review & editing (supporting). **Yamei Peng:** Data curation (supporting); Formal analysis (supporting); Writing‐review & editing (supporting). **Guoxin Zhang:** Data curation (supporting); Validation (supporting); Writing‐review & editing (supporting). **Danxia Zhou:** Data curation (supporting); Writing‐review & editing (supporting). **Yinyin Xie:** Data curation (supporting); Investigation (supporting); Writing‐review & editing (supporting). **Xiaoshuang Hou:** Formal analysis (supporting); Writing‐review & editing (supporting). **Fangfang Zheng:** Formal analysis (supporting); Writing‐review & editing (supporting). **Fan Wang:** Investigation (supporting); Writing‐review & editing (supporting). **Zhenji Gan:** Data curation (supporting); Writing‐review & editing (supporting). **Shuai Chen:** Conceptualization (supporting); Funding acquisition (supporting); Supervision (equal); Writing‐review & editing (equal). **Geng Liu:** Conceptualization (lead); Data curation (lead); Formal analysis (lead); Funding acquisition (lead); Project administration (lead); Supervision (lead); Validation (lead); Writing‐original draft (lead); Writing‐review & editing (lead).

## Supporting information

Supinfo

## Data Availability

Data presented in this manuscript are available upon request from the corresponding authors.
