# Supplementary material for: Increased glycolysis in skeletal muscle coordinates with adipose tissue in systemic metabolic homeostasis
Source: J Cell Mol Med. 2021 Jul 6;25(16):7840–54. doi: 10.1111/jcmm.16698 (PMC8358859; doi:10.1111/jcmm.16698)
Supplement: Supplementary file 1 — Supinfo [file JCMM-25-7840-s001.pdf]

Materials and methods

Mouse generation and animal care

The human *GLUT1* (NM006516), *HK2* (NM000189), and *PFKFB3* (NM004566) full-length ORFs were derived from plasmids from GeneCopoeia (FL24161) and GENECHEM (GC090A06, GC030B01). HA tag, Myc tag, and Flag tag were added to the 5' end of *PFKFB3*, *HK2*, and *GLUT1* ORFs respectively. The three tagged-genes and EGFP cassette were ligated with different virus 2A sequence.[1] To control the expression of transgenes in a temporal- and spatial- specific manner, we constructed an expression plasmid with the polycistronic cassette combined with the Cre-LoxP recombination system and the Tet-On inducible expression system (pRosa-CAG-rtTA-TRE-LSL-HA-PFKFB3-F2A-Myc-HK2-P2A-Flag-GLUT1-T2A-EGFP-WPRE). The linearized pRosa-CAG-rtTA-TRE-LSL-Transgenes vector was homologous recombinated into the *ROSA26* BAC. The linearized *ROSA26*-Transgene BAC was microinjected into the male pronucleus of the zygotes. BAC transgenic mice were created in the Model Animal Research Center of Nanjing University with C57BL/6J background. Genotyping was carried out using primers listed in Table S1. Transgenic copy number estimated by southern blot analysis was performed using standard techniques. The genomic DNA was digested with restriction enzyme *MscI*. A 3' probe, 448 bp in length, was utilized to confirm proper recombination of the long arm (the transgenic and the wild-type ROSA26 loci produced products of 10.3 kb and 15.7 kb, respectively). PCR amplified 3' probes (5'-GGGATGCTTCTGCTTCTGAG-3' and 5'-GCCTTATCTGGAATGGGACA-3') were labeled with DIG. Three Tg lines mice were crossed with the ubiquitous *EIIa-Cre* mouse line initially. The three *EIIA-Cre;Tg* lines mice all exhibited lower body weight and improved glucose tolerance compared with control mice. Line #25 was chosen for further experiments. The *Mck-cre* mice (Jackson Laboratory, stock no. 006475) were backcrossed to C57BL/6J background for at least five generations.

Mice were bred and maintained under specific pathogen-free conditions in a controlled environment of 20-22 ℃, with a 12/12 h light/dark cycle and free access to food and water unless stated. The treatment of 0.2% doxycycline in drinking water was started at the age of 4 to 6 weeks. And no obvious adverse effects were observed during the study. This study was approved by the Institutional Animal Care and Use Committee (IACUC) of Model Animal Research Center, Nanjing University, China, and conducted in accordance with the guidelines of IACUC and the approved Animal protocol #LG19. All the experiments were performed in male mice, except that skeletal muscles from female mice were used for muscle glucose uptake.

Gene expression and protein analysis

Total RNA and protein were extracted and analyzed as previously described.[2, 3] Briefly, total RNA samples were isolated using RNAiso plus (Takara Bio) and were reverse transcribed to cDNA using PrimeScript™ RT reagent kit with gDNA Eraser (Takara Bio) following manufacturer's instructions. Diluted cDNAs were used for Real Time PCR

with SYBR Green reagents (Roche) on an ABI Prism Step-One bio-analyzer. Sequences of primers are listed in Table S2. Expression data were normalized to β-actin mRNA expression. Expression changes were calculated using the ΔΔCt method and expressed as fold change over control. Cells and tissues were harvested, homogenized in ice-cold RIPA buffer with Protease Inhibitor Cocktail Tablets (Roche). Supernatant protein concentration was determined by Pierce™ BCA protein assay kit (Thermo). Proteins were electrophoretically separated and immunoblotted onto polyvinylidene fluoride membranes. Membranes were incubated at 4 °C for 16 h with indicated primary antibodies and subsequently at room temperature for 2 h with horseradish-peroxidase-conjugated secondary antibodies. Detection was carried out with High-sig ECL (Tanon), and signals were visualized by a gel documentation system (Syngene, UK). Protein bands were quantified by densitometry using Image J. The antibodies are listed in Table S3. For immunoprecipitation, tissues were collected and lysed with ice-cold pre-lysis buffer (50 mM Tris-HCl, pH 8.0, 300 mM NaCl, 2 mM EDTA, 1% NP40 with protease inhibitor cocktail). Tissue lysates were incubated with Lamtor1 antibodies (#8975, Cell Signaling) overnight at 4°C on rotation. Protein A/G PLUS-Agarose (sc-2003, Santa Crzu) were then added to the overnight protein aggregates and mixed for another 1h at 4 °C on rotation. The supernatant was collected by centrifugation of the mix at 4 °C, 3000 rpm for 3 min as lysates. The beads were washed with 2x10 minutes with 600 ul of low-salt wash buffer (25 mM Tris-HCl, pH 7.4 and 150 mM NaCl) at 4 °C and another 10 minutes with 600 ul of high-salt wash buffer (25 mM Tris-HCl, pH 7.4 and 500 mM NaCl) at 4 °C. Then the beads were mixed with an equal volume of 2×SDS sample buffer for immunoblotting.

Measurement of oxygen consumption in cells and tissues

Both the ECAR and OCR of MEFs were measured using the XF^e extracellular flux analyzer (Seahorse Bioscience) as follows: 20,000 cells were plated per well into an XF24 cell culture microplate in quintuplicate (5 wells each). For ECAR analysis, final concentrations of 10 mM glucose, 1 μM oligomycin, 100 mM 2-DG (101706-100, Seahorse Bioscience) were applied. And 1 μM oligomycin, 0.5 μM FCCP, 1 μM rotenone and antimycin (102194-100, Seahorse Bioscience) were applied in OCR. Mitochondrial respiration rates were measured in saponin-permeabilized EDL muscle and white fat tissue with indicated substrates as described previously.[4]

Muscle glucose uptake and lipid uptake and oxidation ex vivo

Muscle glucose uptake and lipid uptake and oxidation was carried out in isolated soleus or EDL muscles as previously described.[5, 6] Intact soleus and EDL muscles were isolated from female mice for glucose uptake, and soleus isolated from male mice were applied for lipid uptake and oxidation. Briefly, for glucose uptake, intact soleus and EDL muscles were isolated from mice fasted for 16 hours and incubated with or without insulin in Krebs-Ringer-bicarbonate (KRB) buffer at 37 °C for 50 min. The insulin concentration was 0.1 mU/ml for soleus stimulation and 50 mU/ml for EDL. After incubation, muscles were used for glucose uptake in KRB buffer containing 2-deoxy-D-[1,2-^{3}H(N)] glucose and D-[1-^{14}C] Mannitol for another 10 min in 30 °C with or without insulin. After incubation, the process was terminated in ice-cold KRB buffer containing cytochalasin B, and muscles were blotted dry in liquid nitrogen, weighed, lysed by 1 M NaOH solution at 80

°C, and neutralized by 1 M HCl followed by adding dinonyl-phthalate oil for scintillation counting. ^{3}H and ^{14}C radioisotopes in muscle lysates were measured using a Tri-Carb 2800TR scintillation counter (PerkinElmer) for calculation of muscle glucose uptake. For lipid uptake and oxidation, isolated soleus was stimulated with or without insulin for 30 min and then incubated in KRB buffer (with/without insulin) containing ^{14}C-palmitic acid for another 50 min. After incubation, muscles were blotted dry and lysed for measurements of radioisotopes using a Tri-Carb 2800TR scintillation counter. Gaseous $^{14}CO2$ was evolved from incubation media with 0.6 M perchloric acid (311421, Sigma-Aldrich) and trapped in benzethonium hydroxide-soaked filters (B2156, Sigma-Aldrich). Radioactivity in trapped $^{14}CO2$ was measured to determine muscle lipid oxidation. The sum of radioactivity in muscle and gaseous $^{14}CO2$ was calculated to indicate lipid uptake in muscle.

Blood and tissue analysis

Blood was collected from tail veins of random-fed or overnight-fasted mice. Blood glucose levels were determined using a Breeze 2 glucometer (Bayer). Plasma lactate levels were examined using a Lactate assay kit (A019, Nanjing Jiancheng, China). Plasma NEFA, triglyceride (TG), and total cholesterol (TC) levels were measured using LabAssay NEFA kit (294-63601), LabAssay Triglyceride kit (290-63701) and LabAssay Cholesterol kit (294-65801) (Wako Chemicals USA, Inc.). Plasma insulin levels were determined using an insulin ELISA kit (EZRMI-13K, EMD Millipore, USA). Plasma FGF21 levels were measured by a FGF21 ELISA kit (R&D Systems) according to the manufacturer’s instructions.

Tissue samples were obtained and frozen in liquid nitrogen and then stored at -80 °C until measurement. Tissue TG content was measured following the manufacturer’s instructions (E1013, Applygen). Muscle and cell G-6-P concentrations were determined following the manufacturer’s instructions (MAK014, Sigma). The ATP levels were examined in muscle using a commercially available kit (S0026, Beyotime, China) following the manufacturer’s instructions.

Glucose, insulin, and oral lipid tolerance tests (GTT, ITT, and OLTT)

After withdrawal of food for 16 h (for GTT) or 4 h (for ITT and OLTT), mice were intraperitoneally injected with a bolus of glucose (2 mg glucose per g of body weight) for IPGTT, insulin (0.75 mU insulin per g of body weight) for ITT, or orally administered via gavage with a bolus of olive oil (6 ul olive oil per g of body weight) for OLTT. Blood was collected from tail veins at the indicated times. Blood glucose, plasma free fatty acid and triglyceride levels were determined using Breeze 2 glucometer (Bayer), LabAssay NEFA kit (294-63601), and LabAssay Triglyceride kit (290-63701), respectively.

Assessment of body composition and treadmill endurance

Body weight was measured weekly. Body composition was determined via dual-energy X-ray absorptiometry using a Lunar PIXImus II densitometer (GE Healthcare) following the manufacturer’s

instructions. Mice were acclimated (run for 9 minutes at 10 meters (m)/minute followed by 1 minute at 20 m/minute) to the treadmill for 2 consecutive days prior to the experimental protocol. For low intensity (endurance) exercise studies, fed mice were run for 10 minutes at 10 m/minute followed by a constant speed of 20 m/minute until exhaustion.

Indirect calorimetry

Mice were housed individually in metabolic cages at a 12-h light and dark cycle with free access to food and water using the Comprehensive Lab Animal Monitoring System (CLAMS, Columbus Instruments). Mice were acclimated in the metabolic cage for 1 day prior to the recording according to the instructions of the manufacturer. Food, energy expenditure, physical activity, VO2 and VCO2 were assessed simultaneously.

Metabolomics analysis

LC-HRMS was performed on a Waters UPLC I-class system equipped with a binary solvent delivery manager and a sample manager, coupled with a Waters VION IMS Q-TOF Mass Spectrometer equipped with an electrospray interface (Waters Corporation， Milford, USA). LC Conditions: Column: Acquity BEH C18 column (100 mm × 2.1 mm i.d., 1.7 µm; Waters, Milford, USA). Solvent: The column was maintained at 45 °C and separation was achieved using the following gradient: 5-25% B over 0-1.5 min, 25-100% B over 1.5-10.0 min, 90% B over 10-13 min, the composition was held at 5% B for 2 min at a flow rate of 0.40 mL/min, where B is 10 mM ammonium acetate (PH=9) and A is acetonitrile-10 mM ammonium acetate (PH=9) (v/v=9:1). Injection Volume was 3.00 µL and Column Temperature was set at 45.0 ℃. The mass spectrometric data was collected using a Waters VION IMS Q-TOF Mass Spectrometer equipped with an electrospray ionization source operating in either positive or negative ion mode. The source temperature and de-solvation temperature was set at 120 ℃ and 500 ℃, respectively, with a de-solvation gas flow of 900 L/h. Centroid data was collected from 50 to 1,000 m/z with a scan time of 0.1 s and interscan delay of 0.02 s over a 13 min analysis time.

Cell culture, cell line generation, and RNAi experiments

C2C12 cells (ATCC® CRL-1772™) were seeded into 6-well plate (Corning) 8 h prior to transfection at a density of about 50%. Cells were transfected with pRosa-CAG-rtTA-TRE-Transgene-Neo plasmids using Lipofectamine 3000 transfection reagent (Invitrogen) following the manufacturer's instructions. Twenty-four hours after transfection, the cells were treated with G418 (HyClone) at the final concentration of 400 µg/mL for 10 days for positive selection. Then the cells were picked as clones into 96-well plate. C2C12 cells were cultured with DMEM (Gibco) Supplemented with 10% FBS (Gibco), 50 units/ml penicillin and 50 µg/ml streptomycin under 5% CO2 at 37 ℃ for routine culture. For differentiation assay, C2C12 were cultured to 100% confluence and switched to differentiation medium consisting of DMEM containing 2% horse serum (Gibco) and penicillin/streptomycin for 6 d.

small interfering RNAs (siRNAs) (GenePharma) targeting mouse ATF4 (#1 5’-GGUGGCCAAGCACUUGAAATT-3’, #2 5’-GCUGCUUACAUUACUCUAATT-3’, #3 5’-GCCCAAACCUUAUGACCCATT-3’), MondoA (#1 5’-GCAUGGUAUAUGCAGUAUUTT-3’, #2, 5’-CCCAAUAGCCUCAUUACAUTT-3’, #3, 5’-CCUGCAGCCACAGAAGUUUTT-3’), and ChREBP (#2 5’-GCAGAAGAGGCGGUUCAAUTT-3’, #3 5’-CCAUCACACAUCAGCGCUUTT-3’) were transfected into differentiated myotubes at a final concentration of 100 nM using Lipofectamine 3000 according to the manufacturer’s instructions and were harvested 2-3 days post-transfection.

For co-culture studies, myotubes were washed with PBS and incubated with DMEM for 2 days. CM were then collected, centrifuged at 1,300 g for 5 min, and stored at -80 °C for future use. Primary preadipocytes were isolated and differentiated as previously described.[7] Differentiated adipocytes were treated with CM for 48 h before harvest. For FGF21 blocking studies, CM were incubated with control IgG or anti-FGF21 FL antibodies (#12180, Antibody and Immunoassay Services, HKU) according to the manufacture’s instructions and rotated for 1 h at 4 °C before being applied to adipocytes. For serum neutralization experiments, differentiated adipocytes were treated with DMEM supplied with collected mouse serum for 48 h before analysis. DMEM supplemented with 1% (vol/vol) serum obtained from ctrl and M;G mice were incubated with normal goat IgG or neutralizing antibodies against mouse FGF21 (final concentration, 3 μg/ml; #12180, Antibody and Immunoassay Services, HKU) for 1 h at 4 °C before being applied to adipocytes.

Statistics

Data were expressed as mean ± SEM. Comparisons were performed via unpaired two-tailed student’s t-test for two groups or via two-way ANOVA followed by Bonferroni’s post hoc test for multiple groups using GraphPad Prism 9.0.0 software (GraphPad, San Diego, CA, USA). Comparison of VCO2, VO2, and EE were carried out by ANCOVA with the covariate of body weight using the web-based tool (CalR; https://CalRapp.org/).[8, 9] All p values less than 0.05 were considered statistically significant. Generally, * means $p < 0.05$, ** means $p < 0.01$ and *** means $p < 0.001$. For animal experiments, animal numbers were kept as small as possible, and yet still resulted in statistically meaningful data.

1. Liu Z, Chen O, Wall JBJ, et al. Systematic comparison of 2A peptides for cloning multi-genes in a polycistronic vector. *Scientific Reports*. 2017;7(1):2193. doi:10.1038/s41598-017-02460-2
2. He XY, Xiang C, Zhang CX, et al. p53 in the Myeloid Lineage Modulates an Inflammatory Microenvironment Limiting Initiation and Invasion of Intestinal Tumors. *Cell Rep*. Nov 3 2015;13(5):888-97. doi:10.1016/j.celrep.2015.09.045
3. Chen Q, Rong P, Zhu S, et al. Targeting RalGAPα1 in skeletal muscle to simultaneously improve postprandial glucose and lipid control. *Science Advances*. 2019;5(4):eaav4116. doi:10.1126/sciadv.aav4116
4. Fu T, Xu Z, Liu L, et al. Mitophagy Directs Muscle-Adipose Crosstalk to Alleviate Dietary Obesity. *Cell Rep*. 2018;23(5):1357-1372. doi:10.1016/j.celrep.2018.03.127
5. Chen Q, Rong P, Xu D, et al. Rab8a Deficiency in Skeletal Muscle Causes Hyperlipidemia and Hepatosteatosis by Impairing Muscle Lipid Uptake and Storage. *Diabetes*. 2017;66(9):2387-2399. doi:10.2337/db17-0077
6. Chen S, Wasserman DH, MacKintosh C, Sakamoto K. Mice with AS160/TBC1D4-Thr649Ala knockin mutation are glucose intolerant with reduced insulin sensitivity and altered GLUT4 trafficking. *Cell metabolism*.

2011;13(1):68-79. doi:10.1016/j.cmet.2010.12.005
7. Wu L, Xu D, Zhou L, et al. Rab8a-AS160-MSS4 Regulatory Circuit Controls Lipid Droplet Fusion and Growth. *Developmental Cell*. 2014;30(4):378-393. doi:10.1016/j.devcel.2014.07.005
8. Tschop MH, Speakman JR, Arch JR, et al. A guide to analysis of mouse energy metabolism. *Nat Methods*. Dec 28 2011;9(1):57-63. doi:10.1038/nmeth.1806
9. Mina AI, LeClair RA, LeClair KB, Cohen DE, Lantier L, Banks AS. CalR: A Web-Based Analysis Tool for Indirect Calorimetry Experiments. *Cell Metab*. Oct 2 2018;28(4):656-666.e1. doi:10.1016/j.cmet.2018.06.019

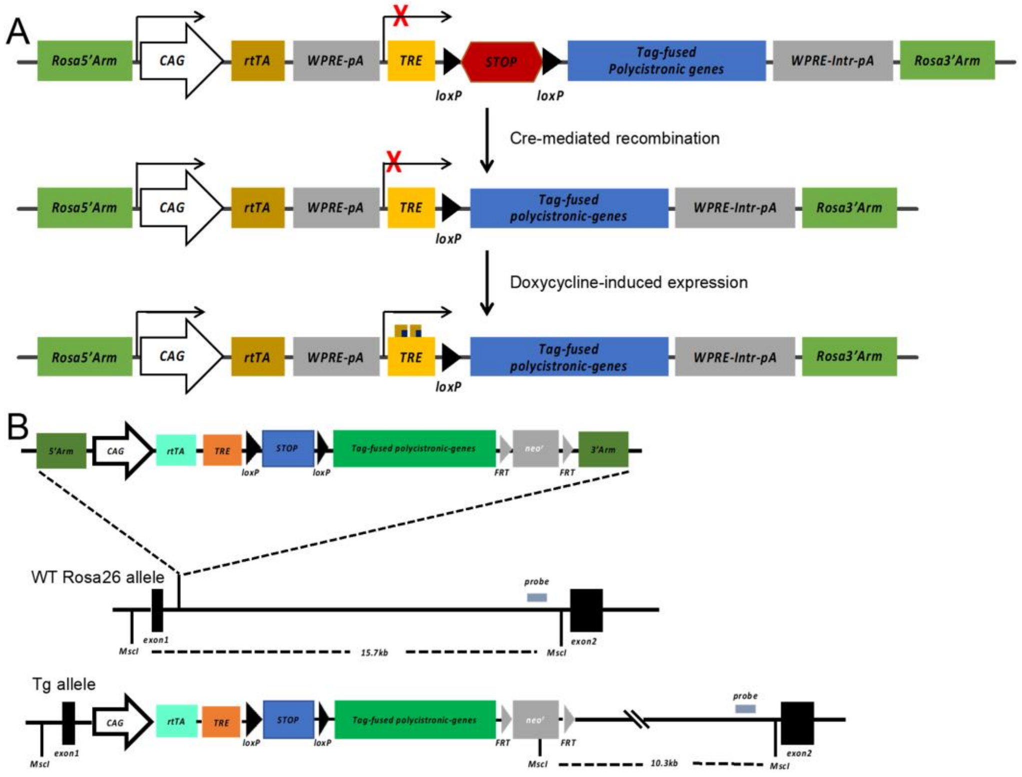

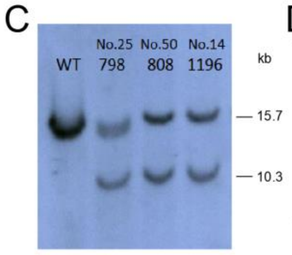

D

No	Photodensity of WT	Photodensity of transgene	Relative photodensity	Copy number
No. 25-798	9690.045	7127.388	0.736	2
No. 50-808	10739.489	8243.731	0.768	2
No. 14-1196	9960.61	8106.338	0.814	2

Figure S1. Generation of BAC transgenic mice overexpressing human *GLUT1*, *HK2*, and *PFKFB3* genes. (A) Schematic representations of the strategy for controlled expression of tag-fused polycistronic genes in Rosa26 BAC transgenic mice. (B) Schematic representations of recombination in the Rosa26 BAC fragment and the identification of transgenic BAC copy numbers. (C) Southern blot analysis of the founder lines. By *MscI*-digestion and 3' probe indication, WT group displayed one band corresponding to 15.7 kb, while *Tg PFKFB3-HK2-GLUT1* group displayed two bands corresponding to 15.7 kb (WT band) and 10.3 kb (*Tg PFKFB3-HK2-GLUT1* band). (D) Quantification of the band density in (C) to estimate the BAC transgene copy number. Values are means ± SEMs, *p < 0.05 (t test).

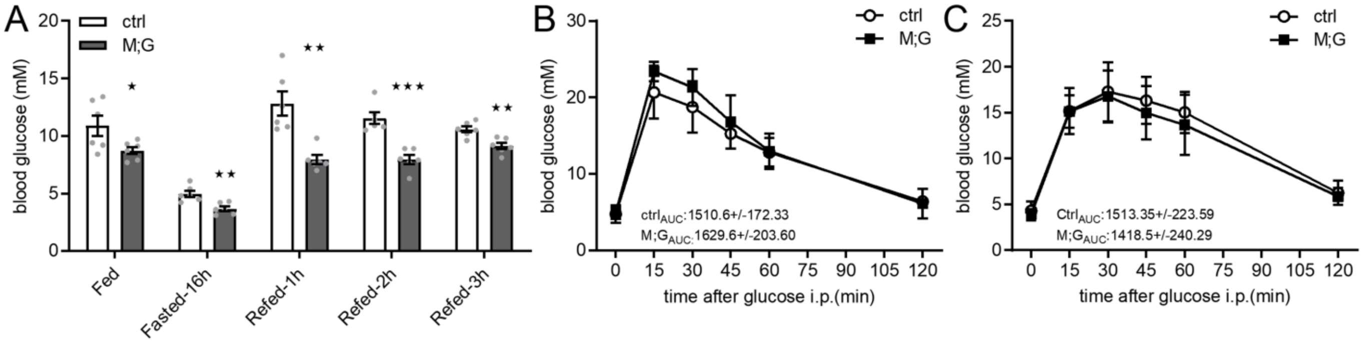

Figure S2. Glucose homeostasis in ctrl and M;G mice. (A) Blood glucose levels in the fed, 16h-fasted, and fasting-refed ctrl and M;G mice. n=6. (B) IPGTT in ctrl and M;G mice without doxycycline-treatment. Data were analyzed two-way ANOVA with Bonferroni's post hoc test. The values showed the glucose area under the curve during GTT. n=7. (C) IPGTT in ctrl and M;G mice after doxycycline-withdrawal. Data were analyzed two-way ANOVA with Bonferroni's post hoc test. The values showed the glucose area under the curve during GTT. n=5 control mice, n=6 M;G mice. Values are means ± SEMs, *$p < 0.05$; **$p < 0.01$; ***$p < 0.001$ (unless stated, data were analyzed via t test).

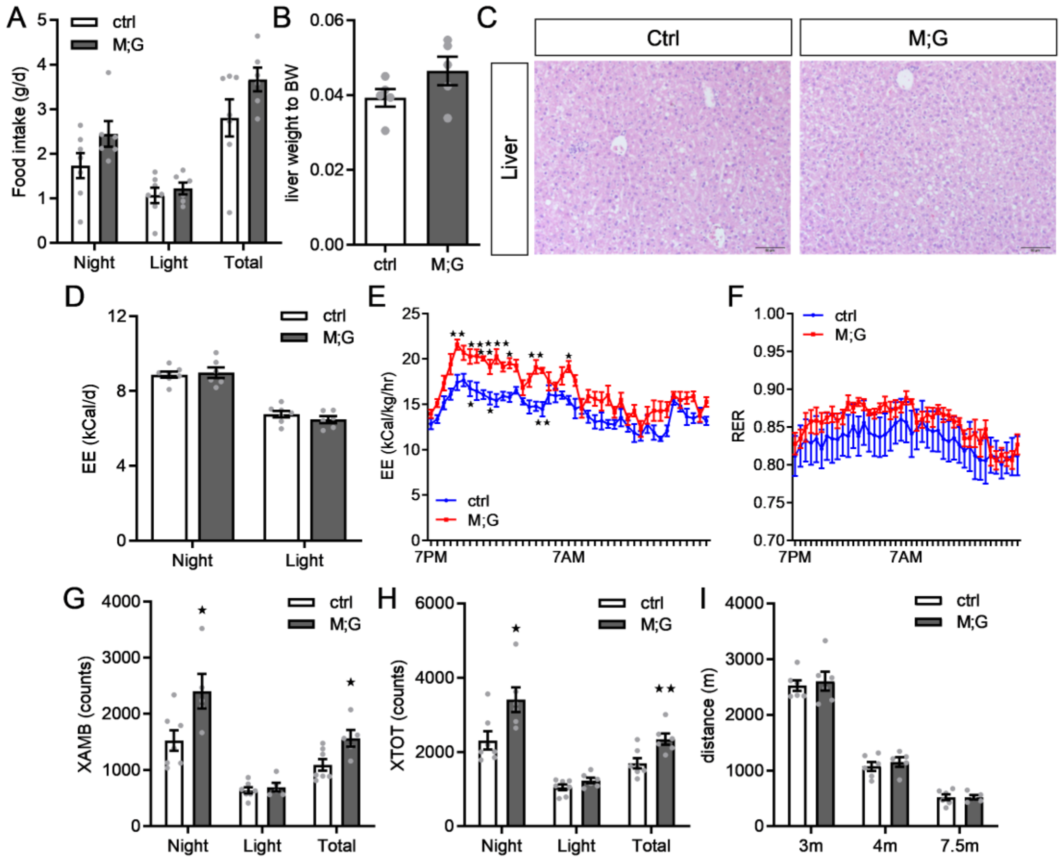

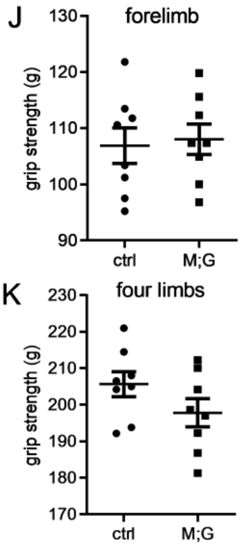

L

	Ctrl	M;G	P
N	6	6	
Body weight (g)	26.20 ± 2.57	27.33 ± 3.01	ns
24 h Food intake (g/d)	4.14 ± 1.41	3.76 ± 0.91	ns
24 h Energy expenditure (kcal/h)	0.42 ± 0.06	0.40 ± 0.06	ns
24 h O_2 consumption (ml/kg/h)	4691.68 ± 588.27	4641.98 ± 765.68	ns
12 h Light O_2 consumption (ml/kg/h)	4431.92 ± 630.55	4369.90 ± 793.72	ns
12 h Night O_2 consumption (ml/kg/h)	4940.14 ± 550.51	4902.23 ± 750.70	ns
24 h CO_2 production (ml/kg/h)	4473.92 ± 731.93	4328.89 ± 862.11	ns
12 h Light CO_2 production (ml/kg/h)	3949.86 ± 701.33	3860.76 ± 826.44	ns
12 h Night CO_2 production (ml/kg/h)	4975.19 ± 764.74	4776.66 ± 902.56	ns
24 h Average RER	0.95 ± 0.05	0.92 ± 0.04	ns
12 h Light RER	0.88 ± 0.04	0.87 ± 0.04	ns
12 h Night RER	1.00 ± 0.06	0.97 ± 0.04	ns
24 h Activity (total counts)	67760 ± 18727	76387 ± 23842	ns
12 h Light activity (total counts)	13747 ± 2639	17745 ± 5579	ns
12 h Night activity (total counts)	54013 ± 19013	58642 ± 20031	ns

Figure S3. Comparable food intake and exercise capacity of ctrl and M;G mice. (A) Food intake of ctrl and M;G mice at the age of 6 months. n=7 control mice, n=6 M;G mice. (B) Relative liver weight of ctrl and M;G mice at the age of 7-8 months. n=5. (C) Histology of liver from ctrl and M;G mice. Scale bar: 50 μm. (D) Energy expenditure in ctrl and M;G mice. n=7 control mice, n=6 M;G mice. Data were analyzed via ANCOVA. (E) Adjust energy expenditure per hour during the light/dark cycle of the mice. The measurements were normalized to mouse body weight. n=7 control mice, n=6 M;G mice. (F) Respiratory exchange ratio per hour during the light/dark cycle of the mice. n=7 control mice, n=6 M;G mice. (G) Ambulatory movement in horizontal axis of the mice at the age of 6 months, average activity over 12 hours light/dark cycle. n=7 control mice, n=6 M;G mice. (H) All movement in horizontal axis of the mice at the age of 6 months, average activity over 12 hours light/dark cycle. n=7 control mice, n=6 M;G mice. (I) Results of forced treadmill exercise test of the 3-month, 4-month and 7.5-month-old mice. 3-month: n=6; 4-month and 7.5-month: n=6 control mice, n=5 M;G mice. (J) Forelimb grip strength of the mice at the age of 4-5 months. n=8. (K) Grip strength of four limbs of the mice at the age of 4-5 months. n=8. (L) Metabolic cage data of the 3-4 months old mice without doxycycline-treatment. Values are means ± SEMs, $*p < 0.05$; $**p < 0.01$; $***p < 0.001$ (unless stated, data were analyzed via t test).

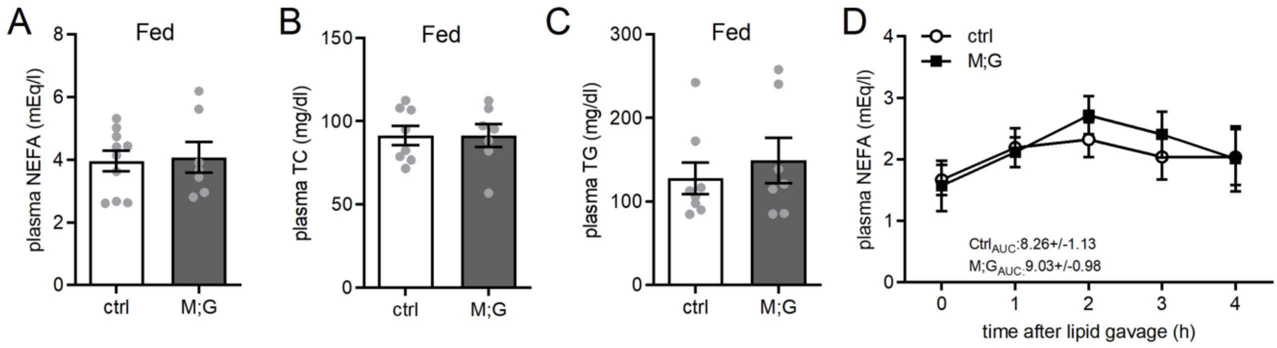

Figure S4. Lipid homeostasis of ctrl and M;G mice. (A-C) Plasma NEFA (A), TC (B), and TG (C) levels in the random-fed mice at 3-4 months of age. n=8 control mice, n=7 M;G mice. (D) Plasma NEFA levels after lipid oral gavage in ctrl and M;G mice at the age of 7 months. Data were analyzed via two-way ANOVA with Bonferroni's post hoc test. The values showed the NEFA aera under the curve during lipid tolerance test. n=7 control mice, n=6 M;G mice. Values are means ± SEMs, (unless stated, data were analyzed via t test).

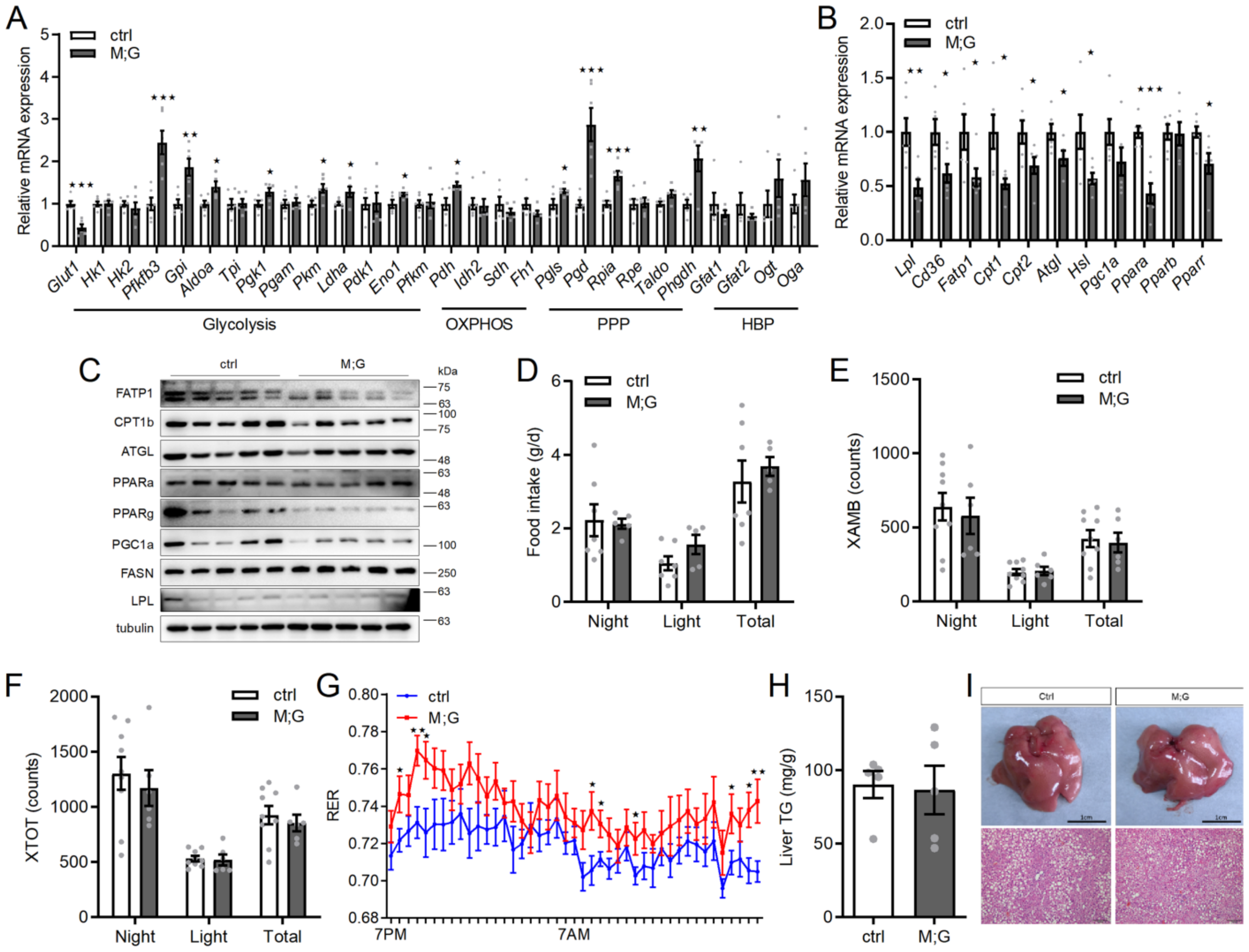

Figure S5. Metabolic profiles of ctrl and M;G mice fed on HFD. (A) Relative mRNA expression of glucose metabolic genes in GC muscle of ctrl and M;G mice fed on HFD. n=6. (B) Relative mRNA expression of lipid metabolic genes in GC muscle of the HFD-fed mice. n=6. (C) Protein expression of lipid metabolic genes in GC muscle from the HFD-fed mice. (D) Food intake of the HFD-fed mice at the age of 3.5-4 months. n=7 control mice, n=5 M;G mice. (E) Ambulatory movement in horizontal axis of the HFD-fed over 12 hours light/dark cycle. n=9 control mice, n=6 M;G mice. (F) All movement in horizontal axis of the HFD-fed mice over 12 hours light/dark cycle. n=9 control mice, n=6 M;G mice. (G) Respiratory exchange ratio per hour during the light/dark cycle of the HFD-fed mice. n=9 control mice, n=6 M;G mice. (H) Liver TG contents in the HFD-fed mice. n=5. (I) Photographs and histology of liver from the HFD-fed mice. Values are means ± SEMs, *$p < 0.05$; **$p < 0.01$; ***$p < 0.001$ (t test).

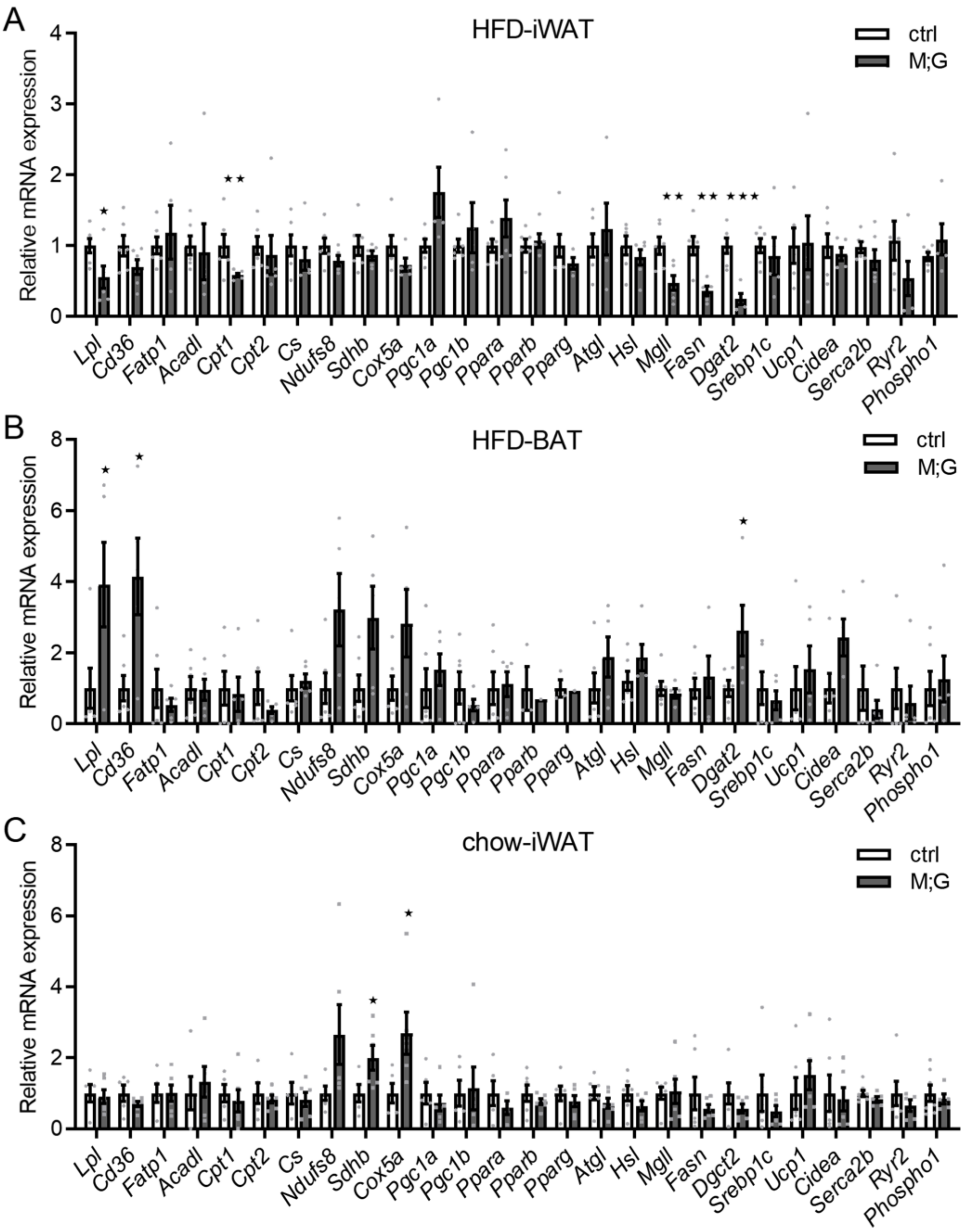

Figure S6. Lipid metabolism in adipose tissues of ctrl and M;G mice. (A) mRNA expression of indicated genes in iWAT from the HFD-fed ctrl and M;G mice. n=6. (B) mRNA expression of indicated genes in BAT from the HFD-fed ctrl and M;G mice. n=6. (C) mRNA expression of indicated genes in iWAT from the chow diet-fed ctrl and M;G mice. n=6. Values are means ± SEMs, *p < 0.05; **p < 0.01; ***p < 0.001 (t test).

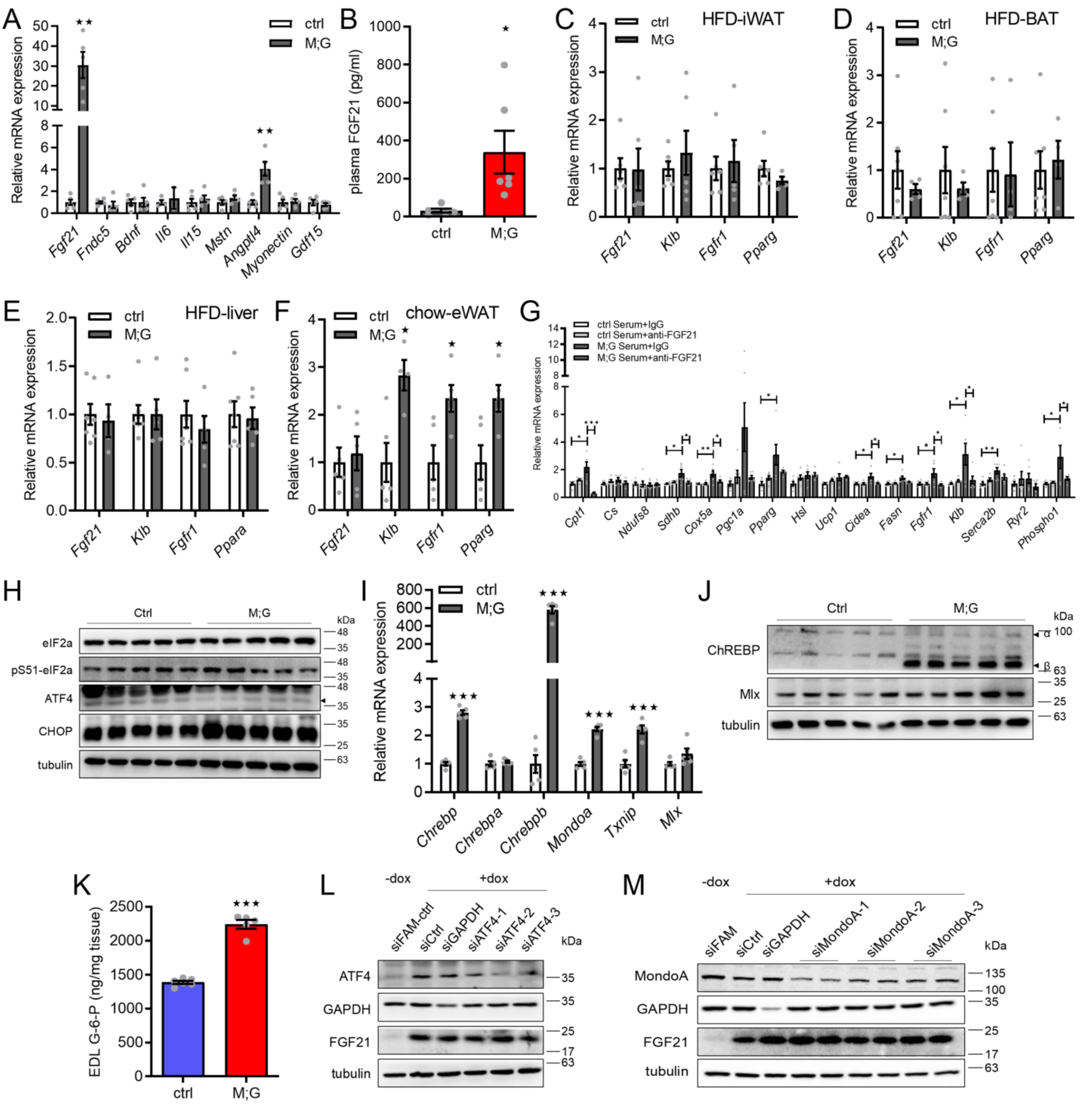

Figure S7. Muscle dependent induction and secretion of FGF21. (A) mRNA expression of indicated myokines in the M;G and ctrl mice fed on chow diet. n=5. (B) Plasma FGF21 levels in the chow diet-fed mice. n=5 control mice, n=6 M;G mice. (C) mRNA expression of indicated genes in iWAT of the HFD-fed mice. n=6. (D) mRNA expression of indicated genes in BAT of the HFD-fed mice. n=6. (E) mRNA expression of indicated genes in liver of the HFD-fed mice. n=6. (F) mRNA expression of indicated genes in eWAT of the mice fed on chow diet. n=5. (G) mRNA expression of indicated genes in the primary visceral white adipocytes treated with serum from either ctrl or M;G mice, with or without the treatment of anti-FGF21 antibody. n=6. (H) Western blot analysis of ATF4 signaling in GC muscle of the HFD-fed mice. Black arrowhead indicates the ATF4 protein. (I) mRNA expression of indicated genes in the M;G and ctrl mice fed on chow diet. n=5. (J) Western blot analysis of ChREBP and Mlx in GC muscle from the mice fed on chow diet. (K) G-6-P levels in EDL muscle of the chow diet-fed mice. n=6 control mice, n=5 M;G mice. (L) Western blot analysis of the 3Genes-OE myotubes transfected with either ATF4-specific or control siRNA, with or without the treatment of doxycycline. FAM-ctrl indicates fluorescein amidites-labeled control siRNA. (M) Western blot analysis of the 3Genes-OE myotubes transfected with either MondoA-specific or control siRNA, with or without the treatment of doxycycline. FAM-ctrl indicates fluorescein amidites-labeled control siRNA. Values are means ± SEMs, $*p < 0.05$; $**p < 0.01$; $***p < 0.001$ (t test).

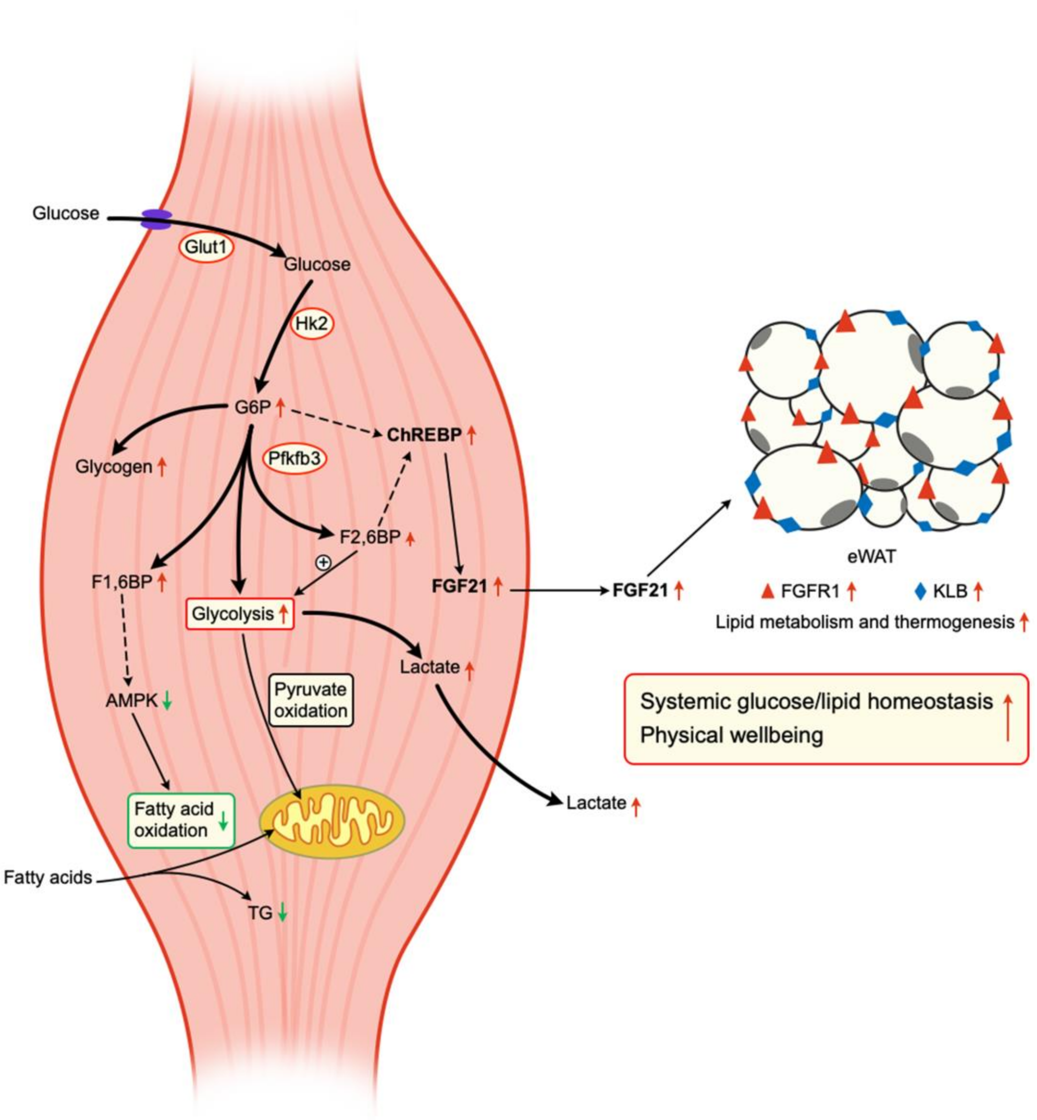

Figure S8. A working model of enhanced skeletal muscle glycolysis in systemic metabolic regulation. The schematic depicts a proposed model for the enhanced anaerobic glycolytic metabolism in skeletal muscle engaging in two inter-connected glucose-sensing pathways to remodel the systemic metabolic network and improve the physical wellbeing.

Table S1. Primer information for genotyping

Genes	Forward primer	Reverse Primer
Mck-cre	5’-GTGAAACAGCATTGCTGTCACTT-3’	5’-TAAGTCTGAACCCGGTCTGC-3’
BAC Tg	5’-GTCGACTGCAGTTAATTAAC-3’	5’-TGCGTCAGTTCCAACGGCATG-3’

Table S2. Primer information for Q-PCR analysis of expression of target genes

Genes	Forward primer	Reverse Primer
b-actin	5'-CCTGTATGCCTCTGGTCGTA-3'	5'-CCATCTCCTGCTCGAAGTCT-3'
18s	5'-GCCGCTAGAGGTGAAATTCTTG-3'	5'-CTTTCGCTCTGGTCCGTCTT-3'
Cpt1	5'-ACTCCTGGAAGAAGAAGTTCA-3'	5'-AGTATCTTTGACAGCTGGGAC-3'
Fatp1	5'-CTACCACTCTGCAGGGAACA-3'	5'-CAGGTAGCGGCAGATTTCAC-3'
Lpl	5'-GGGAGTTTGGCTCCAGAGTTT-3'	5'-TGTGTCTTCAGGGGTCCTTAG-3'
Cd36	5'-CACAGATGCAGCCTCCTTTC-3'	5'-AGCACACCATACGACGTACA-3'
Fabp3	5'-ATCCATGTGCAGAAGTGGAA-3'	5'-CACTGCCATGAGTGAGAGTCA-3'
Atgl	5'-CAACGCCACTCACATCTACGG-3'	5'-TCACCAGGTTGAAGGAGGGAT-3'
Hsl	5'-GGCTCACAGTTACCATCTCACC-3'	5'-GAGTACCTTGCTGTCCTGTCC-3'
Pparα	5'-CCACGAAGCCTACCTGAAGA-3'	5'-TTCTCGGCCATACACAAGGT-3'
Pparβ	5’-TCCATCGTCAACAAAGACGGG-3’	5’-ACTTGGGCTCAATGATGTCAC-3’
Pparγ	5'-TGTGGGGATAAAGCATCAGGC-3'	5'-CCGGCAGTTAAGATCACACCTAT-3'
Pgc1a	5'-CGGAAATCATATCCAACCAG-3'	5'-TGAGAACCGCTAGCAAGTTTG-3'
Glut1	5'-ATGAAAGAAGAGGGTCGGCA-3'	5'-TCCAGCTCGCTCTACAACAA-3'
Glut4	5'-GCCATCGTCATTGGCATTCT-3'	5'-CGCTTTAGACTCTTTCGGGC-3'
Hk1	5'-GAGTCTGAGGTCTACGACACC-3'	5'-CCCACGGGTAATTTCTTGTCC-3'
Hk2	5'-ATGGATGCCTAGATGACTTCCGCA-3'	5'-TAAGTGTTGCAGGATGGCTCGGA-3'
Pfkfb3	5'-AGAACTTCCACTCTCCCACCCAAA-3'	5'-AGGGTAGTGCCCATTGTTGAAGGA-3'
Gpi	5'-TCAAGCTGCGCGAACTTTTTG-3'	5'-GGTTCTTGGAGTAGTCCACCAG-3'
Aldoa	5'-CGTGTGAATCCCTGCATTGG-3'	5'-CAGCCCCTGGGTAGTTGTC-3'
Tpi	5'-CCAGGAAGTTCTTCGTTGGGG-3'	5'-CAAAGTCGATGTAAGCGGTGG-3'
Pgk1	5'-ATGTCGCTTTCCAACAAGCTG-3'	5'-GCTCCATTGTCCAAGCAGAAT-3'
Pgam	5'-TCTGTGCAGAAGAGAGCAATCC-3'	5'-CTGTCAGACCGCCATAGTGT-3'
Ldha	5'-TGTCTCCAGCAAAGACTACTGT-3'	5'-GACTGTACTTGACAATGTTGGGA-3'
Pdk1	5'-GGCGGCTTTGTGATTTGTAT-3'	5'-ACCTGAATCGGGGGATAAAC-3'
Eno	5'-TGCGTCCACTGGCATCTAC-3'	5'-CAGAGCAGGCGCAATAGTTTTA-3'
Pdh	5'-AGGAGGGAATTGAATGTGAGGT-3'	5'-ACTGGCTTCTATGGCTTCGAT-3'
Idh2	5'-GGAGAAGCCGGTAGTGGAGAT-3'	5'-GGTCTGGTCACGGTTTGGAA-3'
Sdh	5'-GCTGCGTTCTTGCTGAGACA-3'	5'-ATCTCCTCCTTAGCTGTGGTT-3'
Fh1	5'-GAATGGCAAGCCAAAATTCCTT-3'	5'-CGTTCTGTAGCACCTCCAATCTT-3'
Pgls	5'-CCAGGTCCTTACCATCAATCCT-3'	5'-AGGGAAGAGCGAACAGGTATG-3'
Pgd	5'-ATGGCCCAAGCTGACATTG-3'	5'-GCACAGACCACAAATCCATGAT-3'
Rpia	5'-AAGGCCGAGGAGGCTAAGAA-3'	5'-CTTTCAGCTATTCGCTGCACA-3'
Rpe	5'-GCACCTGGATGTAATGGACGG-3'	5'-CCTGGCCTAGCTGCTTTCG-3'
Taldo	5'-GTAAAGCGCCAGAGGATGGAG-3'	5'-CTCTTGGTAGGCAGGCATCT-3'
Ppgdh	5'-ATGGCCTTCGCAAATCTGC-3'	5'-AGTTCAGCTATCAGCTCCTCC-3'
Gys1	5'-CCGGCTTTGGCTGCTTTAT-3'	5'-CCGATCCAGAATGTAAATGCC-3'
Ugp2	5'-ACCTGGGATACCTGCCGTG-3'	5'-CCTGCTCACCCCTTCCTTC-3'
Gbe1	5'-ACACCAGGGAAGTTCAAAATTGTAC-3'	5'-GTGTTGTGGTCCAGTCTCTGATG-3'
Ppp1r1a	5'-ACGGAAGAAGATGACAAGGACC-3'	5'-TTGCCCTAGGTGATGTTCAACC-3'
G6pase	5'-TTAAAGAGACTGTGGGCATCAAT-3'	5'-ATCCACTTGAAGACGAGGTTG-3'
Pepck-c	5'-CCATCACCTCCTGGAAGAACAAG-3'	5'-ACCCTCAATGGGTACTCCTTCTG-3'
Chrebp	5’-CACTCAGGGAATACACGCCTAC-3’	5’-ATCTTGGTCTTAGGGTCTTCAGG-3’
Chrebpα	5’-CGACACTCACCCACCTCTTC-3’	5’-TTGTTCAGCCGGATCTTGTC-3’
Chrebpβ	5’-TCTGCAGATCGCGTGGAG-3’	5’-CTTGTCCCGGCATAGCAAC-3’
MondoA	5’-TGCTACCTGCCACAGGAGTC-3’	5’-GACTCAAACAGTGGCTTGATGA-3’
Mlx	5’-CATGGACTCCCTCTTCCAGTC-3’	5’-TGATGAAGGACACCGATCACA-3’
Fgf21	5’-TTCTTTGCCAACAGCCAGAT-3’	5’-GTCCTCCAGCAGCAGTTCTC-3’
Fgfr1	5’-TTCATCCTGGTGGTGGCAGC-3’	5’-GCTCTTCTTGGTGCCGCTCT-3’
Klb	5’-GATGAAGAATTTCCTAAACCAGGTT-3’	5’-AACCAAACACGCGGATTTC-3’
Serca2b	5’-ACCTTTGCCGCTCATTTTCC-3’	5’-AGGCTGCACACACTCTTTAC-3’
Ryr2	5’-ATGGCTTTAAGGCACAGCG-3’	5’-CAGAGCCCGAATCATCCAGC-3’
Phospho1	5’-AGCTGGAGACCAACAGTTTC-3’	5’-TCCCTAGATAGGCATCGTAGT-3’
mt-Nd1	5’-CCCATTCGCGTTATCTT-3'	5’-AAGTTGATCGTAACGGAAGC-3'

Table S3. The list of antibodies used in this study

Antibody name	Company	Cat No.
anti-HA	Sigma	H9658
anti-Flag	Sigma	F3165
anti-c-Myc	Santa Cruz	sc-40
anti-GFP	Santa Cruz	sc-8334
anti-HK2	Santa Cruz	sc-6521
anti-PFKFB3	Abcam	ab181861
anti-GLUT1	Abcam	ab652
anti-CPT1	Abcam	ab15703
anti-LPL	Proteintech	16899-1-AP
anti-G6PD	Proteintech	66373-1-AP
anti-p70S6K	Proteintech	14485-1-AP
anti-mTOR	Cell Signaling Technology	#2983
anti-pS2448-mTOR	Cell Signaling Technology	#5536
anti-pT389-p70S6K	Cell Signaling Technology	#9234
anti-pS371-p70S6K	Cell Signaling Technology	#9208
anti-4EBP1	Cell Signaling Technology	#9644
anti-pT37/46-4EBP1	Cell Signaling Technology	#2855
anti-AKT	Cell Signaling Technology	#9272
anti-pS473-AKT	Cell Signaling Technology	#9271
anti-pT308-AKT	Cell Signaling Technology	#3038
anti-GSK3	Cell Signaling Technology	#5676
anti-pS21/9-GSK3	Cell Signaling Technology	#9331
anti-TSC2	Cell Signaling Technology	#3990
anti-pS939-TSC2	Cell Signaling Technology	#3615
anti-IR	Cell Signaling Technology	#3025
anti-ACC	Cell Signaling Technology	#3676
anti-pS79-ACC	Cell Signaling Technology	#3661
anti-AMPK	Cell Signaling Technology	#2532
anti-pT172-AMPK	Cell Signaling Technology	#2531
anti-FASN	Cell Signaling Technology	#3180
anti-PPARγ	Cell Signaling Technology	#2435
anti-O-GlcNAc	Cell Signaling Technology	#9875
anti-ATGL	Merck Millipore	ABD66
anti-PGC1α	Merck Millipore	AB3242
anti-AS160	Merck Millipore	07-741
anti-pT649-AS160	Life Technologies	441071G
anti-CD36	Santa Cruz	sc-9154
anti-PPARα	Santa Cruz	sc-9000
anti-Mlx	Santa Cruz	Sc-393086
anti-ChREBP	Novus	NB400-135
anti-MondoA	Proteintech	13614-1-AP
anti-FGF21	R&D Systems	AF3057
anti-ATF4	Abcam	Ab216839
anti-CHOP	Cell Signaling Technology	#2895
anti-eIF2α	Cell Signaling Technology	#5324
anti-pS51-eIF2α	Cell Signaling Technology	#3398
anti-GAPDH	Bioworld	AP0063
anti-aTubulin	Abclonal	AC012
HRP Bovine anti-goat IgG	Santa Cruz	sc-2352
HRP Goat anti-Rabbit IgG	Invitrogen	31460
HRP Goat anti-mouse IgG	Invitrogen	62-6520
